# Residual Beta Cell Function in Newly Diagnosed Type 1 Diabetes after Treatment with Atorvastatin: The Randomized DIATOR Trial

**DOI:** 10.1371/journal.pone.0017554

**Published:** 2011-03-11

**Authors:** Stephan Martin, Christian Herder, Nanette C. Schloot, Wolfgang Koenig, Tim Heise, Lutz Heinemann, Hubert Kolb, on behalf of the DIATOR Study Group

**Affiliations:** 1 Institute for Clinical Diabetology, German Diabetes Center, Leibniz Center for Diabetes Research at Heinrich Heine University, Düsseldorf, Germany; 2 Departments of Medicine and Metabolic Diseases, University Hospital, Düsseldorf, Germany; 3 Department of Internal Medicine II - Cardiology, University of Ulm Medical Center, Ulm, Germany; 4 Profil Institute for Metabolic Research, Neuss, Germany; Brigham & Women's Hospital, and Harvard Medical School, United States of America

## Abstract

**Background:**

Recent evidence suggests that the lipid-lowering agent atorvastatin is also a potent immunomodulator. The aim of this study was to investigate the possible effect of atorvastatin on the decline of residual beta cell function in recent-onset type 1 diabetes.

**Methods and Findings:**

The randomised placebo-controlled Diabetes and Atorvastatin (DIATOR) Trial included 89 patients with newly diagnosed type 1 diabetes and islet autoantibodies (mean age 30 years, 40% females), in 12 centres in Germany. Patients received placebo or 80 mg/d atorvastatin for 18 months. As primary outcome stimulated serum C-peptide levels were determined 90 min after a standardized liquid mixed meal. An intent-to-treat analysis was performed. Fasting and stimulated C-peptide levels were not significantly different between groups at 18 months. However, median fasting serum C-peptide levels dropped from baseline to 12 and 18 months in the placebo group (from 0. 34 to 0.23 and 0.20 nmol/l, p<0.001) versus a nonsignificant decline in the atorvastatin group (from 0.34 to 0.27 and 0.30 nmol/l, ns). Median stimulated C-peptide concentrations declined between baseline and 12 months (placebo from 0.89 to 0.71 nmol/l, atorvastatin from 0.88 to 0.73 nmol/l, p<0.01 each) followed by a major loss by month 18 in the placebo group (to 0.48 nmol/l, p = 0.047) but not in the atorvastatin group (to 0.71 nmol/l, ns). Median levels of total cholesterol and C-reactive protein decreased in the atorvastatin group only (p<0.001 and p = 0.04). Metabolic control was similar between groups.

**Conclusions:**

Atorvastatin treatment did not significantly preserve beta cell function although there may have been a slower decline of beta-cell function which merits further study.

**Trial Registration:**

ClinicalTrials.gov NCT00974740

## Introduction

Immunosuppressive treatment of recent onset type 1 diabetes has been shown to slow the decline of residual beta cell function [1]. Recent trials which reported a delay in disease progression include autologous stem cell therapy, treatment with immunomodulatory monoclonal antibodies or vaccination with disease-associated autoantigens (see ref.2). The latter approach did not cause recognizable treatment-related adverse effects. Statins have been considered as immunomodulary agents because of their ability to suppress the expression of adhesion molecules and MHC class II molecules as well as of inflammatory mediators such as C-reactive protein [3], [4]. The inhibition of T-cell activation involves the blockade of the interaction between T-lymphocytes and antigen presenting cells by binding to an adhesion molecule involved in this process, LFA-1 [5]. Soluble forms of ICAM-1 – the natural receptor of LFA-1- were found to be decreased in recent onset type 1 diabetic patients [6] and to inhibit type 1 diabetes specific autoantigen T-cell proliferation [7]. Furthermore, administration of recombinant forms of soluble ICAM-1 was effective in inhibiting insulitis and diabetes-development in NOD mice [8].

Atorvastatin showed beneficial effects in patients with rheumatoid arthritis [9], and in relapsing-remitting multiple sclerosis [10]. Another trial reported an increase of disease activity for the combination of atorvastatin with interferon-β [11] whereas one subsequent trial did not find such an adverse effect [12]. A third trial reported better outcomes for the combination of atorvastatin with interferon-β [13].

The possible beneficial effect of statin therapy on the beta cell destructive process in pancreatic islets has been analysed in animal models, with inconsistent results. In the multiple low-dose streptozotocin models in CD-1 mice, administration of simvastatin delayed or protected from the development of insulin-deficient diabetes [14], whereas no effect was seen with atorvastatin treatment in C57BL/6 mice [15]. Statin treatment lowered the incidence of diabetes in the autoimmune diabetic NOD mouse model in one out of three studies [15]–[17] Treatment with simvastatin prolonged survival of islets transplanted to NOD mice [14], [18].

In view of the disease modifying activity of statins in two human immune-mediated diseases we initiated the DIATOR (Diabetes and Atorvastatin) Trial investigating the effects of treatment with atorvastatin in the course of recent-onset type 1 diabetes.

## Results

**Figure 1:**
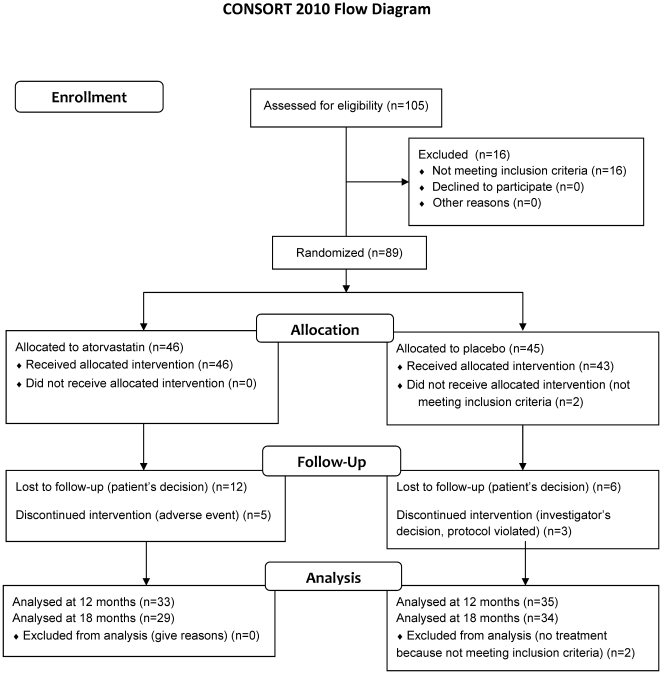
Study flow diagram. Patients were screened in 12 participating centers by using the inclusion and exclusion criteria.

During the years 2004–2006 eighty-nine of the 105 patients with recent-onset type 1 diabetes screened were identified as eligible. Despite an extension of the recruitment period and of the number of participating centers the goal of 160 patients was not reached. The decision to stop screening was made by the Study Committee based on the low recruitment rate of the last 12 months, while still being blinded for patient allocation to treatment groups. After randomization two patients in the placebo group were excluded because of not having met the inclusion criteria of at least one islet autoantibody, leaving 87 patients for the intent-to-treat analysis. In total, 78% of patients completed the visit at 12 months and 72% the final visit at 18 months (**Fig. 1**).

### Baseline Characteristics

The two study groups were comparable with respect to baseline parameters (**Table 1**). There also were no significant differences between study centers, or between patients completing the full study and drop-outs at different time points, with the exception of higher baseline insulin dose in drop-outs vs. completers (not shown).

**Table 1:** Baseline characteristics.

Characteristic	Atorvastatin Group	Placebo Group
Mean age (SD), y	30.0 (6.8)	29.8 (6.5)
Men, n (%)	26 (57	27 (63)
Mean BMI (kg/m^2^) (SD)	23.4 (3.1)	24.2 (3.0)
Mean HbA1c (%) (SD)	7.8 (1.8)	7.5 (1.6)
Mean insulin dose (IU/kg) (SD)	0.32 (0.18)	0.33 (0.19)
Median fasting C-peptide (nmol/l)(IQR)	0.34 (0.22)	0.34 (0.20)
Median stim. C-peptide (nmol/l)(IQR)^a^	0.88 (0.66)	0.89 (0.67)
GAD65 autoantibodies^b^:		
patients positive (n)	42	40
median titer (U/ml) (IQR)	34 (63)	21 (46)
IA-2 autoantibodies^b^:		
patients positive (n)	25	25
median titer (U/ml) (IQR)	8.6 (16)	6.3 (13)
Islet cell antibodies^b^		
patients positive (n)	37	30
median titer (JDFU/ml) (IQR)	40 (0)	40 (0)
Patients positive for one autoantibody (n)	7	8
Patients positive for two autoantibodies (n)	14	12
Patients positive for three autoantibodies (n)	23	21

a Stimulated serum C-peptide concentrations were determined 90 min after intake of a standardized liquid mixed meal.

b Data on GAD, IA2 and islet cell antibodies were not available from 2, 6 and 4 persons, respectively.

### Main Outcomes

**Figure 2:**
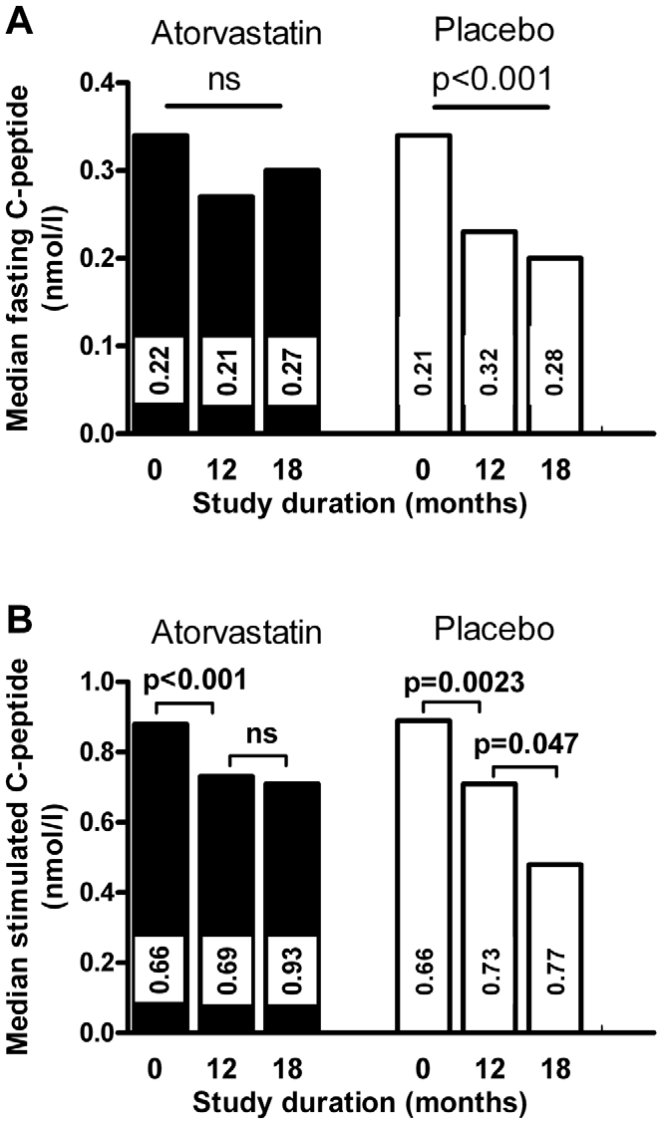
Course of C-peptide secretion in the study groups. Fasting serum C-peptide concentrations (**a**) were the mean of two blood samples obtained 5 min apart, samples for stimulated serum C-peptide (**b**) were obtained 90 min after a standard liquid mixed meal. Numbers in columns indicate the IQR. Filled bars, atorvastatin, empty bars, placebo. Differences between groups were not significant. Comparison between groups by Mann-Whitey-u-test, within groups by Wilcoxon signed rank test.

Median fasting and stimulated C-peptide concentrations did not differ significantly between atorvastatin and placebo at 18 months (0.30 vs. 0.20 nmol/l, p = 0.40, and 0.71 vs. 0.48 nmol/l, p = 0.36, respectively) although there was a 50% difference for fasting and a 48% difference for stimulated C-peptide. The course of C-peptide secretion over the study period is depicted in **Fig. 2**. Median fasting serum C-peptide levels dropped from baseline to 12 and 18 months in the placebo group (from 0. 34 to 0.23 and 0.20 nmol/l, p<0.001) whereas they remained stable in the atorvastatin group (from 0.34 to 0.27 and 0.30 nmol/l, ns) (**Fig. 2A**). Mixed-meal stimulated beta cell secretion initially decreased in both groups until 12 months (from 0.89 to 0.71 nmol/l in the placebo group, from 0.88 to 0.73 nmol/l in the atoravastatin group, p<0.01 for both) with no further deterioration until 18 months in the atorvastatin group (0.71 nmol/l), whereas there was significant further loss of beta cell function in the placebo group (0.48 nmol/l, p<0.046, **Fig. 2B**). Additional exploratory analyses were performed to search for a treatment effect in subgroups defined by center allocation, high vs. low age, BMI or baseline levels of fasting or stimulated C-peptide, number of islet autoantibodies. No significant difference for the primary endpoint at 18 months was found.

### Efficacy of Atorvastatin Treatment

**Figure 3:**
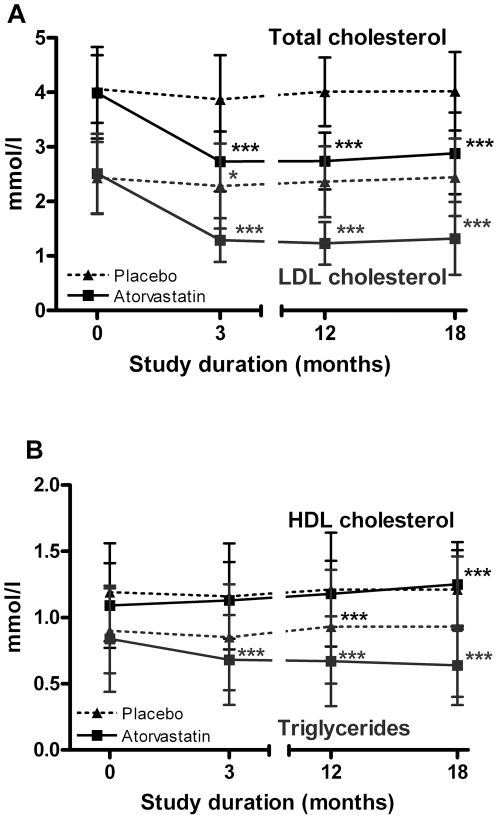
Effect of atorvastatin on blood lipid levels. Shown are mean levels of total (black lines) and LDL-cholesterol (blue lines) (**a**), HDL-cholesterol (black lines) and triglycerides (blue lines) (**b**), bars indicate standard deviation, dotted lines depict placebo, continuous lines atorvastatin. * p<0.05, ** p<0.01, *** p<0.001 for comparison with baseline values. Only changes for the atorvastatin group are significant.

In the atorvastatin group median baseline concentrations of total cholesterol (4.04 mmol/l), LDL-cholesterol (2.51 mmol/l) and triglyceride (0.75 mmol/l) decreased by 3 months and remained at low levels throughout the treatment period (**Fig. 3**). From baseline to 18 months decreases were 32.2% for total, 52.3% for LDL-cholesterol, and 26.0% for triglyceride concentrations. (p<0.001, each). Median HDL-cholesterol levels increased from 1.05 mmol/l at baseline to 1.22 mmol/l at 18 months (p<0.001). In the placebo group, there were no significant changes throughout the treatment period.

Changes in immune parameters were determined by comparing serum concentrations at baseline and 3 months. Median plasma CRP concentrations decreased slightly in the atorvastatin (from 0.95 (IQR 2.01) to 0.73 mg/l (1.03), p = 0.03, but not in the placebo group (from 0.88 (1.59) to 0.78 mg/l (1.31)). No significant changes were observed in either group for median plasma concentrations of the soluble adhesion molecules sICAM-1 and E-selectin, or serum concentrations of cytokines IFNγ, IL-6, IL-18 cytokine antagonist IL-1ra, chemokines eotaxin, IP-10, MCP-4, MIP-1β, MDC, IL-8 and TARC (data not shown). Median concentrations of MCP-1 decreased significantly in the placebo group (from 431 to 356 pg/ml, p = 0.009) but not in the atorvastatin group (from 367 to 303 pg/ml, ns).). The only difference between groups was a 11% higher concentration of IL-1ra at 3 months with atorvastatin treatment (p = 0.02).

### Metabolic Control

**Figure 4:**
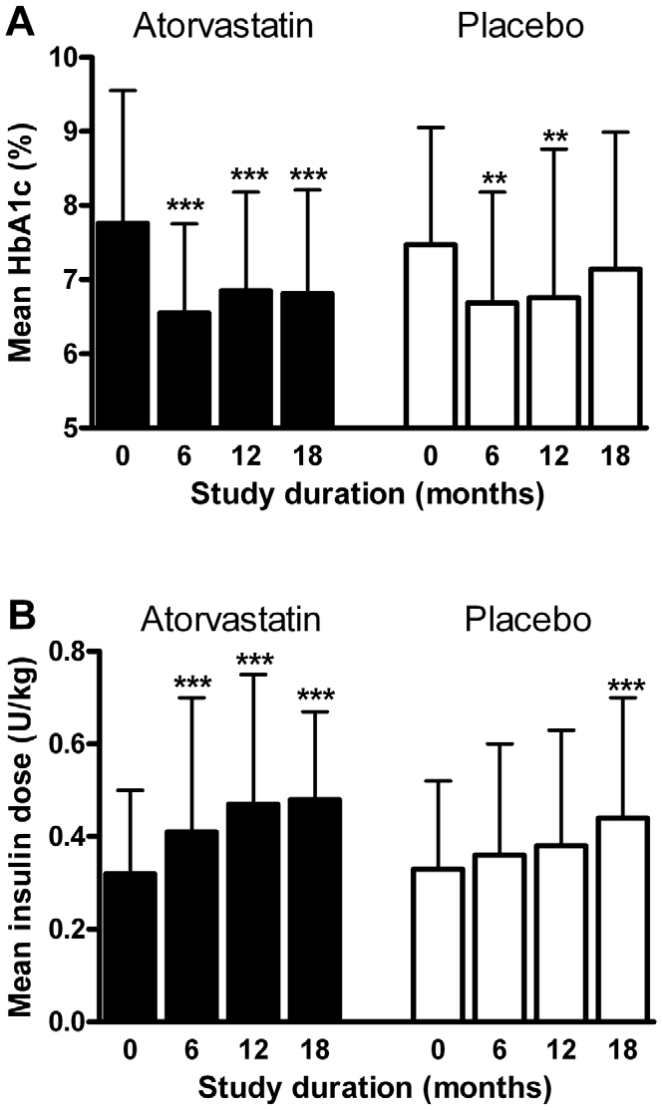
Metabolic control during the study (HbA1c (a), insulin dose (b). Filled bars, atorvastatin, empty bars, placebo. Bars indicate standard deviation, for HbA1c p-values describe differences of mean values from baseline determined by ANCOVA with correction for baseline HbA1c and BMI (which correlated with later HbA1c levels), **, p<0.01; ***, p<0.001. Mean insulin doses per kg bodyweight were different between groups at 12 months (p = 0.007, comparison of log-transformed mean values by ANCOVA with correction for baseline insulin dose and age (which correlated with later insulin doses).

Mean HbA1c levels decreased from baseline to 6 months in both study groups (from 7.8 to 6.6% with atorvastatin, from 7.5 to 6.7% with placebo, both p<0.001) and stayed at a lower level throughout the treatment period in the atorvastatin group (6.8% at 18 months), while under placebo treatment mean values increased and were no more different from baseline at 18 months (7.1%, p>0.05) (**Fig. 4A**). However, differences between treatment groups were not significant at either 12 or 18 months. Mean daily insulin dose increased in the atorvastatin group from 0.32 IU/kg at baseline to 0.48 IU/kg at 18 months, and in the placebo group from 0.33 to 0.44 IU/kg (both p<0.001). The rise in insulin dose was more rapid in the atorvastatin group, resulting in a higher dose at 12 months compared to the placebo group, p = 0.007 (**Fig. 4B**).

### Safety Results

Dose reductions (from 80 mg/d to 40 mg/d) due to adverse effects occurred for 1–1.7 years in 3 cases: 1 case of myalgia and myopathia (atorvastatin), 1 of arthralgia (placebo) and 1 of increased CPK serum levels (atorvastatin). Medication was temporarily discontinued in 7 cases because of symptoms lasting between 1 and 28 days (atorvastatin: Helicobacter infection, bilious colic in patient with gallstone predisposition, swollen lids with headache, back pain after moving into a new domicile, respiratory infection with fever; placebo: muscle pain, blood in stool) Permanent discontinuation of atorvastatin occurred in three persons, after temporary treatment halt because of intestinal cramps, because of mildly elevated hepatic enzyme levels or because of neurological symptoms. In the atorvastatin group, 18 patients (39.1%) reported 64 adverse events vs. 15 patients (34.9%) with 31 adverse events in the placebo group. In the atorvastatin group 9 of the 64 adverse events in 7 patients were rated as “possibly” or “probably” related to medication, vs. 4 of the 28 adverse events in 4 placebo patients (difference not significant). Five events in 4 patients of the atorvastatin group and 3 events in 3 patients of the placebo group were classified as severe adverse events; none was classified as “possibly” or “probably” related to medication. All patients recovered or were stabilized. CPK levels were elevated in 16 patients of the atorvastatin and in 6 patients of the placebo group. However, in most cases these elevations were of mild character (maximum 405 U/l in the atorvastatin vs. 321 U/l in the placebo group) and critical serum levels of >10 times of the upper normal range (i.e. >2000 U/l) were never observed.

## Discussion

The trial did not find a significant effect of atorvastatin treatment with regard to the primary endpoint, i.e. the comparison of stimulated C-peptide concentrations between groups at 18 months. Also, the two groups did not differ significantly with regard to fasting C-peptide levels. In both cases, median C-peptide concentrations were around 50% higher in the atorvastatin than the placebo group at 18 months, but this difference failed to be significant because of an unexpectedly large range or standard deviation of C-peptide concentrations measured, in both groups. In this regard, a major limitation of the trial is that the actual number of patients recruited was lower than foreseen in the study protocol (total 89 vs. 160).

As secondary analysis we compared median C-peptide concentrations over the study period within a group. There was a significant deterioration of residual beta cell function in the placebo group but not the atorvastatin group. In the placebo group, median fasting C-peptide concentrations decreased from baseline by 32% at 12 months and by 41% at 18 months (p<0.001), whereas there was no significant change in the atorvastatin group. Median stimulated C-peptide concentrations decreased mildly in both groups by 12 months (by 20% in the placebo group and by 17% in the atorvastatin group, p<0.01 each). At 18 months median C-peptide concentrations had further decreased (p = 0.046), total decrease by 46% of baseline, whereas there was no further deterioration in the atorvastatin group, total decrease by 19%.

These differences were not reflected by lower insulin doses in the atorvastatin group. Rather, there was a more rapid increase of insulin dosing leading to significantly higher insulin doses at 12 months although not at 18 months. Some types of statins have been reported to increase or decrease insulin resistance but a consistent effect was not noted for atorvastatin in a recent meta-analysis [19]. Partial preservation of residual beta cell function is considered as clinically relevant goal also in the absence of lower insulin requirements, because of a lower risk of complications [20], [21].

Since beta cell function is affected by the concentration of glucose at the start of the test, the study protocol requested that no mixed meal test should be performed if fasting blood glucose was outside 4–11 mmol/l. However, even in this concentration range, ambient glucose may affect the outcome of beta cell function tests, as suggest by the recent international workshop comparing the liquid mixed meal with the glucagon assay [22]. There was an inverse relationship between fasting blood glucose and subsequent peak C-peptide concentrations following the liquid mixed meal. This means that the higher HbA1c levels (and consequently higher mean fasting blood glucose levels) in the atorvastatin group at 12 and 18 months probably has led to a lower C-peptide response, and hence has downsized the difference between the two groups. Unfortunately, there is no algorithm or formula for adjusting C-peptide responses for ambient glucose.

As expected, regular intake of atorvastatin caused a decrease of total and LDL-cholesterol levels in serum, a decrease of triglyceride levels and a small increase of HDL-cholesterol levels. Median concentrations of CRP were low at baseline, but were further lowered by statin treatment. All of these effects are consequences of inhibiting the synthesis of mevalonate from acetyl coenzyme A by hydroxyl-3-methylglutaryl-coenzyme A reductase. This is a rate limiting step in cholesterol synthesis, and the mevalonate pathway gives rise to a number of compounds such as farnesyl or geranylgeranyl pyrophosphate which can modify several transcription factors controlling cell growth, endothelial activity and immune gene expression [3], [23]–[26]. The amelioration of the lipid status by atorvastatin treatment may be considered advantageous, independent of the slowed loss of beta cell function. The decrease in systemic CRP levels may indicate a dampening of inflammatory processes, although our analysis of circulating concentrations of 14 different immune mediators, including soluble adhesion molecules, cytokines, a cytokine antagonist and chemokines did not reveal statin-treatment associated changes. This argues against a major effect on systemic immune reactivity but does not exclude that statin treatment affects islet-antigen specific cellular immunity, e.g. through an increase of regulatory T-cell functions in pancreatic islets or draining lymph nodes. Indeed, statin blockade of the mevalonate pathway has been observed to promote the generation of Foxp3 positive regulatory T-cells in mice and a shift from autoaggressive Th1 to more benign Th2 immunity [27]–[30]. Atorvastatin-induced Krüppel-like factor 2 expression may be a critical event for these effects [31]. Besides the immunomodulatory properties atorvastatin may also target pancreatic islets. A recent study in streptozotocin treated rat pups reported an increase in the number of small islets following atorvastatin treatment, suggestive of neogenesis. Since angiogenesis preceded the increase in beta cell mass, target of atorvastatin may be the endothelium [32].

Atorvastatin was generally well tolerated. Dose reductions or temporary discontinuation of treatment were reported in 8 cases, in three cases treatment was permanently discontinued. Elevation of CPK levels were observed more often in the atorvastatin than in the placebo group (35 vs 14%) but did not reach critical levels of >2000 U/l.

In summary, we report that treatment with atorvastatin over 18 months was safe and well tolerated in adult patients with recent-onset type 1 diabetes. At 18 months, the atorvastatin group did not exhibit significantly higher fasting or stimulated C-peptide concentrations than the placebo group. Secondary analyses of the course of C-peptide secretion within groups found some preservation of fasting and stimulated serum C-peptide concentrations in the atorvastatin but not the placebo group. A comparison with results from the ongoing trial of atorvastatin in children and adolescents with type 1 diabetes (ClinicalTrials.gov registration number NCT00529191) will help to judge the potential of this treatment modality.

## Methods

### Ethics statement

The study was conducted in accordance with the Declaration of Helsinki, and approval by the ethics committees of the Ärztekammer Nordrhein was obtained. All patients provided written informed consent prior to study entry. The protocol for this trial and supporting CONSORT checklist are available as supporting information; see Checklist S1 and Protocol S1.

### Study population

In participating centers throughout Germany (n = 12), patients with newly diagnosed type 1 diabetes were screened for eligibility. In accordance with current guidelines this new treatment modality was first tried in adult patients. Inclusion criteria were insulin requiring diabetes for two weeks to 3 months, tested positive for at least one islet autoantibody (to glutamic acid decarboxylase (GAD) 65, to insulinoma-associated antigen (IA)-2 or islet cell antibodies (ICA)), age 18–39 years. Female patients were to use contraceptive methods. Major exclusion criteria were concomitant other diseases, use of anti-inflammatory, antihypertensive, lipid-lowering or antidiabetic drugs other than insulin, a serum creatine phosphokinase (CPK) level >5 times the upper limit of normal, a serum LDL-cholesterol level >150 mg/dl or any other conditions considered relevant by the investigator. All patients were of Caucasian ethnicity.

### Procedure

The study was a Phase I trial conducted as randomized, double-blind, placebo-controlled, outpatient, parallel group study. Patients were assigned to treatment with either atorvastatin or placebo for a period of 18 months in a one-to-one manner using a computer-generated randomization list, with stratification for participating centers. The starting atorvastatin dose was 40 mg/d or a matching number of placebo tablets. After 4 weeks, the dose was increased to 80 mg/d or matching placebo. Counts of unused tablets were performed and did not indicate a lack of compliance, defined by >20% of tablets returned on two consecutive visits. During the study, the investigators were advised to reduce the dose to 40 mg/d in case of side-effects (e.g. myalgia). Insulin treatment was continued throughout the study with a recommended treatment goal of HbA1c below 6.5% from 3 months on.

### Follow-up and Outcome Measures

Patients attended visits at 0, 3, 6, 12 and 18 months, without food intake for the preceding 10 hours. Primary outcome was stimulated C-peptide secretion, assessed as serum C-peptide concentrations after a standardised liquid mixed meal (Boost HP® (Mead Johnson, Evansville, IN, USA), 6 ml per kg body weight with a maximum of 360 ml) which was performed at months 0, 12 and 18. Patients had to refrain from alcohol intake and unaccustomed strenuous physical activity for 48 hours prior to the test. In the morning, a capillary blood glucose measurement was done and the test was performed if fasting blood glucose was ≥4 mmol/l and ≤11.1 mmol/l. The test was performed in the morning between 7 and 10 a.m. The patient should not have taken any short-acting insulin at least 6 hours prior to the test. A first blood sample was drawn five minutes, a second one immediately before the liquid meal was taken in (mean value as formal “time zero”). The test meal had to be ingested within 5 minutes. Another blood sample was drawn after 90 min, following the guideline of the Diabetes Control and Complications Trial [33]. A recent international workshop found a good correlation between measuring C-peptide levels at this single time point versus determining the area under the curve [22]. Primary outcome measure was stimulated serum C-peptide levels at 12 or 18 months. Serum glucose, C-peptide (immunoenzymatic assay, Biosource/Invitrogen, Karlsruhe, Germany), lipid and immune parameters were analyzed in a central laboratory.

C-reactive protein (CRP) concentrations were determined by an immunonephelometric assay [34], all other immune mediators were measured by double-antibody ELISA (sICAM-1, E-selectin, IL-6, IL-1ra, IFNγ, eotaxin, IP-10, MCP-4, MIP-1β, MDC, TARC) or bead-based multiplex technology (IL-18, MCP-1, IL-8) [35]. GAD65 and IA-2 antibodies were determined by radioligand binding assay (CentAK anti-GAD65, CentAK anti-IA-2, Medipan, Berlin, Germany). ICA was determined by immunofluorescent staining on human AB- positive pancreatic sections as described [36]. The immune laboratory participated in Standardization Workshops DASP 2009 of the International Diabetes Society with following results: GAD antibody sensitivity 0.72; specificity 0.91; IA-2 antibody sensitivity 0.64, specificity 0.96.

### Statistical Analysis

Based on data on C-peptide levels from patient files of the German Diabetes Center it was calculated that for the primary endpoint 80 patients per group would allow to recognize a 20% difference between study groups in median stimulated serum C-peptide levels at 18 months with a type 1 error of <1%, power 95%. For the actual number of patients recruited, the p-value was <0.05, power 80%. All patients who received at least one dose of study treatment were included in the safety analysis. The statistical evaluation (intent-to-treat analysis) was performed by using validated software (SAS Version 9.2). Tests for normal distribution were performed by Kolmogorov-Smirnov. Values with (log-)normal distribution are given as mean/SD, comparisons between groups were made with unpaired t-test or ANOVA, or by paired t-test within groups; F-test was used to check whether or not variances were equal and appropriate tests were used: pooled variance test if variances were equal, Satterthwaite test if variances were unequal. Comparisons between groups included all patients, for comparisons within a group only those patients could be included where baseline and later data were available. Values without normal distribution are given as median and interquartile range (compared between groups with Mann-Whitney u-test, within groups with Wilcoxon signed rank test) Dichotomous/categorical variables are given as proportions (Fisher's exact or Chi-square test). Missing data were not substituted. Exploratory analyses were performed for patient subgroups below or above median age, BMI, stimulated or fasting C-peptide at baseline.

## Supporting Information

Checklist S1 (DOC)

Protocol S1 (PDF)

## References

[1] The Canandian/European Randomized Control Trial Group (1988). Cyclosporin-induced remission of IDDM after early intervention.
Association of 1 yr of cyclosporin treatment with enhanced insulin
secretion. The Canadian-European Randomized Control Trial
Group.. Diabetes.

[2] Waldron-Lynch F, Herold KC (2009). Advances in Type 1 diabetes therapeutics: immunomodulation and
beta-cell salvage.. Endocrinol Metab Clin North Am.

[3] Blank N, Schiller M, Krienke S, Busse F, Schatz B (2007). Atorvastatin inhibits T cell activation through
3-hydroxy-3-methylglutaryl coenzyme A reductase without decreasing
cholesterol synthesis.. J Immunol.

[4] Bonnet J, McPherson R, Tedgui A, Simoneau D, Nozza A (2008). Comparative effects of 10-mg versus 80-mg Atorvastatin on
high-sensitivity C-reactive protein in patients with stable coronary artery
disease: results of the CAP (Comparative Atorvastatin Pleiotropic effects)
study.. Clin Ther.

[5] Weitz-Schmidt G, Welzenbach K, Brinkmann V, Karnata T, Kallen J (2001). Statins selectively inhibit leukocyte function antigen-1 by
binding to a novel regulatory integrin site.. Nat Med.

[6] Lampeter ER, Kishimoto TK, Rothlein R, Mainolfi EA, Bertrams J (1992). Elevated levels of circulating adhesion molecules in IDDM
patients and in subjects at risk for IDDM.. Diabetes.

[7] Roep BO, Heidenthal E, de Vries RR, Kolb H, Martin S (1994). Soluble forms of intercellular adhesion molecule-1 in
insulin-dependent diabetes mellitus.. Lancet.

[8] Martin S, Heidenthal E, Schulte B, Rothe H, Kolb H (1998). Soluble forms of intercellular adhesion molecule-1 inhibit
insulitis and onset of autoimmune diabetes.. Diabetologia.

[9] McCarey DW, McInnes IB, Madhok R, Hampson R, Scherbakov O (2004). Trial of Atorvastatin in Rheumatoid Arthritis (TARA):
double-blind, randomised placebo-controlled trial.. Lancet.

[10] Paul F, Waiczies S, Wuerfel J, Bellman-Strobl J, Dorr J (2008). Oral high-dose atorvastatin treatment in relapsing-remitting
multiple sclerosis.. PLoS One.

[11] Birnbaum G, Cree B, Altafullah I, Zinser M, Reder AT (2008). Combining beta interferon and atorvastatin may increase disease
activity in multiple sclerosis.. Neurology.

[12] Rudick RA, Pace A, Rani MR, Hyde R, Paanzara M (2009). Effect of statins on clinical and molecular responses to
intramuscular interferon beta-1a.. Neurology.

[13] Lanzillo R, Orefice G, Quarantelli M, Rinaldi C, Prinster A (2010). Atorvastatin combined to interferon to verify the efficacy
(ACTIVE) in relapsing-remitting active multiple sclerosis patients: a
longitudinal controlled trial of combination therapy.. Mult Scler.

[14] Rydgren T, Vaarala O, Sandler S (2007). Simvastatin protects against multiple low-dose
streptozotocin-induced type 1 diabetes in CD-1 mice and recurrence of
disease in nonobese diabetic mice.. J Pharmacol Exp Ther.

[15] Palomer X, Calpe-Berdiel L, Verdaguer J, Carillo J, Pastor X (2005). Atorvastatin does not decrease or delay diabetes onset in two
different mouse models of type 1 diabetes.. Diabetologia.

[16] Lozanoska-Ochser B, Barone F, Pitzalis C, Peakman M (2006). Atorvastatin fails to prevent the development of autoimmune
diabetes despite inhibition of pathogenic beta-cell-specific CD8
T-cells.. Diabetes.

[17] Zhang S, Yan X, Zhou PC (2008). [Effects of pravastatin in prevention of diabetes and
mechanism thereof: experiment with non-obese diabetic
mice].. Zhonghua Yi Xue Za Zhi.

[18] Contreras JL, Smyth CA, Bilbao G, Young CJ, Thompson JA, Eckhoff DE (2002). Simvastatin induces activation of the serine-threonine protein
kinase AKT and increases survival of isolated human pancreatic
islets.. Transplantation.

[19] Baker WL, Talati R, White CM, Coleman CI (2010). Differing effect of statins on insulin sensitivity in
non-diabetics: a systematic review and meta-analysis.. Diabetes Res Clin Pract.

[20] Kolb H, Gale EA (2001). Does partial preservation of residual beta-cell function justify
immune intervention in recent onset Type I diabetes?. Diabetologia.

[21] Palmer JP, Fleming GA, Greenbaum CJ, Herold KC, Jansa LD (2004). C-peptide is the appropriate outcome measure for type 1 diabetes
clinical trials to preserve beta-cell function: report of an ADA workshop,
21-22 October 2001.. Diabetes.

[22] Greenbaum CJ, Mandrup-Poulsen T, McGee PF, Batllino T, Haastert B (2008). Mixed-meal tolerance test versus glucagon stimulation test for
the assessment of beta-cell function in therapeutic trials in type 1
diabetes.. Diabetes Care.

[23] Kwak B, Mulhaupt F, Myit S, Mach F (2000). Statins as a newly recognized type of
immunomodulator.. Nat Med.

[24] Arnaud C, Burger F, Steffens S, Veillard NR, Nguyen TH (2005). Statins reduce interleukin-6-induced C-reactive protein in human
hepatocytes: new evidence for direct antiinflammatory effects of
statins.. Arterioscler Thromb Vasc Biol.

[25] Blum A, Shamburek R (2009). The pleiotropic effects of statins on endothelial function,
vascular inflammation, immunomodulation and thrombogenesis.. Atherosclerosis.

[26] Montecucco F, Burger F, Pelli G, Poku NK, Berlier C (2009). Statins inhibit C-reactive protein-induced chemokine secretion,
ICAM-1 upregulation and chemotaxis in adherent human
monocytes.. Rheumatology (Oxford).

[27] Kim YC, Kim KK, Shevach EM (2010). Simvastatin induces Foxp3+ T regulatory cells by modulation
of transforming growth factor-beta signal transduction.. Immunology.

[28] Youssef S, Stuve O, Patarroyo JC, Ruiz PJ, Radosevich JL (2002). The HMG-CoA reductase inhibitor, atorvastatin, promotes a Th2
bias and reverses paralysis in central nervous system autoimmune
disease.. Nature.

[29] Hakamada-Taguchi R, Uehara Y, Kuribayashi K, Numabe A, Saito K, Negoro H (2003). Inhibition of hydroxymethylglutaryl-coenzyme a reductase reduces
Th1 development and promotes Th2 development.. Circ Res.

[30] Dunn SE, Youssef S, Goldstein MJ, Prod'homme T, Weber MS (2006). Isoprenoids determine Th1/Th2 fate in pathogenic T cells,
providing a mechanism of modulation of autoimmunity by
atorvastatin.. J Exp Med.

[31] Bu DX, Tarrio M, Grabie N, Zhang Y, Yamazaki H (2010). Statin-induced Kruppel-like factor 2 expression in human and
mouse T cells reduces inflammatory and pathogenic responses.. J Clin Invest.

[32] Marchand KC, Arany EJ, Hill DJ (2010). Effects of atorvastatin on the regeneration of pancreatic
{beta}-cells after streptozotocin treatment in the neonatal
rodent.. Am J Physiol Endocrinol Metabol.

[33] The Diabetes Control and Complications Trial Research Group (1998). Effect of intensive therapy on residual beta-cell function in
patients with type 1 diabetes in the diabetes control and complications
trial. A randomized, controlled trial.. Ann Intern Med.

[34] Herder C, Peltonen M, Koenig W, Sutfels K, Lindstrom J (2009). Anti-inflammatory effect of lifestyle changes in the Finnish
Diabetes Prevention Study.. Diabetologia.

[35] Herder C, Baumert J, Thorand B, Koenig W, de Jager W (2006). Chemokines as risk factors for type 2 diabetes: results from the
MONICA/KORA Augsburg study, 1984-2002.. Diabetologia.

[36] Hanifi-Moghaddam P, Schloot NC, Kappler S, Seissler J, Kolb H (2003). An association of autoantibody status and serum cytokine levels
in type 1 diabetes.. Diabetes.

