# Supplementary material for: Residual Beta Cell Function in Newly Diagnosed Type 1 Diabetes after Treatment with Atorvastatin: The Randomized DIATOR Trial
Source: PLoS One. 2011 Mar 11;6(3):e17554. doi: 10.1371/journal.pone.0017554 (PMC3055882; doi:10.1371/journal.pone.0017554)
Supplement: Protocol S1 — (PDF) [file pone.0017554.s002.pdf]

Study Protocol

A Randomized, Double-Blind, Placebo-Controlled, Parallel Group Study to Investigate the Effect of Atorvastatin on Residual Beta-Cell Function and Glycemic Control in Patients with Newly Diagnosed Type 1 Diabetes Mellitus

DIATOR
Diabetes Intervention with Atorvastatin

Investigational product:	Atorvastatin
Indication:	Newly Diagnosed Type 1 Diabetes Mellitus
Study Committee	Stephan Martin, Hubert Kolb, Werner A. Scherbaum
Principal Investigator	Stephan Martin
Version:	2.3
Date:	07 September 2003

Signature Page

I confirm that I will work according to this protocol. I will also work consistently with the ethical principles that have their origin in the Declaration of Helsinki and that are consistent with good clinical practices and the applicable laws and regulations. I will accept the monitor's overseeing of the study. I will promptly submit the protocol to applicable ethical review board(s). I confirm that if I or any of my staff are members of the ethical review board, we will abstain from voting on this protocol.

Signatures of the Study Committee

Prof. Dr. med. Stephan Martin
Principal Investigator

Signature Date

Prof. Dr. rer. nat. Hubert Kolb

Signature Date

Prof. Dr. Werner Scherbaum

Signature Date

Synopsis

Title	A Randomized, Double-Blind, Placebo-Controlled, Parallel Group Study to Investigate the Effect of Atorvastatin on Residual Beta-Cell Function and Glycemic Control in Patients with Newly Diagnosed Type 1 Diabetes Mellitus<br><br>**DIATOR: Diabetes Intervention with Atorvastatin**
Investigational product:	Atorvastatin
Objectives:	1. To assess the effect of atorvastatin on pancreatic beta-cell function as measured by C-peptide after a liquid mixed meal stimulation in patients with newly diagnosed type 1 diabetes mellitus<br>2. To assess the effect on metabolic control as measured by HbA1c and insulin requirements<br>3. To assess the safety and tolerability of atorvastatin in the treatment of newly diagnosed type 1 diabetes patients as indicated by adverse events, changes in physical examination, ECG, vital signs, and changes in laboratory parameters that are relevant to safety<br>4. To assess the effect on risk factors of diabetic complications as indicated by changes in serum lipids and CRP in patients with newly diagnosed type 1 diabetes<br>5. To assess the effect on systemic immune abnormalities in patients with type 1 diabetes mellitus as measured by effects on beta-cell autoantibodies, blood cytokines and chemokines on protein and transcriptional level
Number of patients:	Up to 160 patients with recent onset type 1 diabetes mellitus should complete all study procedures. Patients who are withdrawn from the study during the first nine months of treatment will be replaced.
Number of study sites:	About 20 study sites will participate in this trial. The number of sites may be adjusted to assure a sufficient number of patients can be included.<br>The German Diabetes Clinic will participate with approximately 25 to 35 patients.

Efficacy endpoints:	*Primary efficacy endpoint*:<br>• Area under the C-peptide curve in a 2 hour mixed meal tolerance test after 12 months and 18 months of treatment<br><br>*Secondary efficacy endpoint*:<br>• HbA1c and insulin requirements after 12 and 18 months of treatment
Study drug regimen:	starting dose 40 mg/day for 4 weeks, then increase to 80 mg/day, in case of side effects reduction to 40 mg/day<br>Frequency: daily, as recommended<br>Route: oral<br>Duration: 18 months

Table of contents

1 List of Abbreviations 9

2 Investigators and Study Administrative Structure 10
2.1 Requirements of the Investigator(s) and Study Sites 10
2.2 Administrative Structure 10

3 Introduction 12

4 Study Objectives 14

5 Overall Study Design and Procedures 15
5.1 Study Design 15
5.2 Study Flow Chart 17
5.3 Study Procedures 18
5.3.1 Screening Visit 18
5.3.2 Visit 1 18
5.3.3 Visit 2 19
5.3.4 Visit 3 20
5.3.5 Visit 4 20
5.3.6 Visit 5 20
5.3.7 Early Discontinuation Visit 21

6 Selection of Study Population 22
6.1 Primary Diagnosis 22
6.2 Number of Subjects 22
6.3 Inclusion Criteria 22
6.4 Exclusion Criteria 22
6.5 Withdrawal Criteria 23

7 Treatments to be Administered 25
7.1 Identity of Investigational Product 25
7.2 Labeling and Packaging of the Investigational Product 25
7.3 Drug Accountability 25
7.4 Blinding 26
7.5 Randomization 26
7.6 Treatment Compliance 26

8 Study Assessments 27
8.1 Demographic Data 27
8.2 Medical History, including Concomitant Illnesses 27
8.3 Concomitant Medication, including insulin requirements 27
8.4 Body weight, Height and BMI 27
8.5 Vital Signs 28
8.6 Physical Examination 28
8.7 12-Lead ECG 28
8.8 Safety Laboratory 28
8.8.1 Hematology 28
8.8.2 Clinical chemistry 28

8.8.3 Urinalysis....28
8.8.4 Coagulation....29
8.9 Serum Lipids....29
8.10 Pregnancy Test....29
8.11 HbA1c and Fasting C-peptide....29
8.12 Beta-cell Autoantibodies....29
8.13 Plasma CRP....29
8.14 Serum Cytokines and Serum Chemokines....30
8.15 Transcriptional Analysis....30
8.16 Two Hour Mixed Meal Tolerance Test....30
8.17 Adverse Events....31
8.17.1 Definition of Adverse Events....31
8.17.2 Definition of Serious Adverse Events....32
8.17.3 Change of Laboratory Parameters....33
8.17.4 Documentation and Reporting of Adverse Events....33

9 Statistical Evaluation....35
9.1 Determination of sample size....35
9.2 Statistical and Analytical Plan....35
9.3 Demographics and Baseline Medical Characteristics....35
9.4 Definition of Study Populations for Analysis....35
9.5 Handling of Missing and Incomplete Data....35
9.6 Safety Evaluation....36
9.7 Evaluation of Immune Response....36
9.8 Evaluation of Clinical Efficacy....36
9.9 Interim Analyses....36

10 Ethics....38
10.1 Independent Ethics Committee....38
10.2 Ethical Conduct of the Study....38
10.3 Patient Information and Consent....38

11 Documentation, Record Access and Archiving....39
11.1 Documentation of Patients' Participation....39
11.2 Documentation of Essential Documents/Supplements at Study Site During the Trial....39
11.3 Patients' Records....39
11.4 Case Report Forms....40
11.5 Archiving....40

12 Quality Assurance and Quality Control....40
12.1 Monitoring....40
12.2 Data Management/Coding....41
12.3 Audit and Supervision of the Study....41

13 Administrative Procedures....42
13.1 Amendments to the Protocol....42
13.2 Disclosure of all Information and Results....42
13.3 Premature Discontinuation of the Study in a Trial Site....43
13.4 Premature Discontinuation of the Complete Study....43
13.5 Insurance for Patients....43

13.6 Contracts, Finances 43
14 Reporting 44
15 References 45

1 LIST OF ABBREVIATIONS

ADA	American Diabetes Association
ALT	Alanine aminotransferase
ANOVA	Analysis of variance
AP	Alkaline phosphatase
AST	Aspartate aminotransferase
BMI	Body mass index
CD	Cluster of differentiation
CPK	Creatine phosphokinase
CPMP	Committee for Proprietary Medicinal Products
CRF	Case report/record form
CRP	C-reactive protein
ECG	Electrocardiogram
GAD	Glutamic acid decarboxylase
GCP	Good Clinical Practice
GGT	Gamma glutamyl transferase
HbA1c	Glycosylated hemoglobin
HCG	Human chorionic gonadotropin
HDL	High-density lipoprotein
HMG-CoA	Hydroxy-methylglutaryl Coenzyme A
IA-2	Tyrosine phosphatase (Autoantibodies)
ICA	Islet cell antibody
ICAM-1	Intracellular adhesion molecule
ICH	International Conference on Harmonisation
IFN	Interferon
IL	Interleukin
INR	International normalised ratio (for reporting prothrombin time)
LDH	Lactate dehydrogenase
LDL	Low-density lipoprotein
LFA-1	lymphocyte function-associated antigen-1
MCH	Mean corpuscular hemoglobin
MCHC	Mean corpuscular hemoglobin concentration
MCP-1	Monocyte chemotactic protein-1
MCV	Mean corpuscular volume
MHC	Major histocompatibility complex
MIP-1	Monocyte inflammatory protein-1
MMTT	mixed meal tolerance test
NOD	Nonobese diabetic
PTT	Partial Thromboplastin Time
SOP	Standard Operating Procedure
Th1	T helper cell subset 1
Th2	T helper cell subset 2
Treg	Regulatory T cell

2 INVESTIGATORS AND STUDY ADMINISTRATIVE STRUCTURE

2.1 Requirements of the Investigator(s) and Study Sites

The study will take place on an out-patient basis at about 20 study sites in Germany. The number of participating sites may be adjusted, if necessary to ensure a sufficient number of patients can be included.
Investigators will be familiar with the requirements and obligations of Good Clinical Practice. Investigators and site personnel will undergo study specific training during the site initiation.

2.2 Administrative Structure

Principal Investigator:	*Prof. Dr. Stephan Martin*<br>*German Diabetes Clinic*<br>*D-40225 Düsseldorf*
Study Committee:	*Prof. Dr. Stephan Martin*<br>*Prof. Dr. Hubert Kolb*<br>*Prof. Dr. Werner A. Scherbaum*<br>*German Diabetes Clinic*<br>*D-40225 Düsseldorf*
Coordinating Center	*Profil Institute for Metabolic Research GmbH*<br>*D-41460 Neuss*
Safety Review Board:	two independent outside expert and a representative of Profil GmbH. All members of the Safety Review Board will be physicians who will otherwise not be involved in the conduction of the study. The members of the Safety Review Board will be appointed prior to study initiation.

This study is supported by an academic grant from Pfizer GmbH, D-76032 Karlsruhe

The German Diabetes Clinic will participate with approximately 25 to 35 patients.

Profil GmbH will serve as a coordinating center and will be responsible for
- study monitoring
- participation in site selection
- training and supporting the investigators and site staff in all study related procedures
- supplying sites with study medication and study materials
- labeling, packaging, storage, shipping and disposal of study medication
- randomization procedures

- design, distribution, review and archiving of CRF-documents
- participation in the external Safety Review Board
- data management
- quality assurance
- implementation of a service as a “Central Telephone Call-Center” for the study sites
- collection of adverse events
- 24-hour-hotline for reporting of severe adverse events.

The Safety Review Board will consist of three physicians (two outside experts and a Profil representative). Informal safety analysis will be performed 6-monthly or more frequently, if required. The Safety Review Board will take part in an interim analysis after 40 percent of all participants will have completed twelve months of treatment. Randomization code will only be broken to investigators, if necessary for reasons of safety. Decisions will be taken on the discretion of the Safety Review Board and the Study Committee .Decisions will be protocolled. Any changes to the protocol as a result of the data review will be made as amendments to the protocol.

3 INTRODUCTION

Type 1 Diabetes mellitus results from a selective destruction of the insulin secreting beta-cells of the pancreas, caused by a T-cell-mediated autoimmune process. Progressive destruction of beta-cells leads to hyperglycemia due to a lack of insulin. An imbalance in the T-cell immunoregulatory abilities plays a key role in the progression of the disease. Beta-cell destruction is enhanced by the T-helper-1 (Th1) subset of the $CD4^+$ T cells and their cytokines while the T-helper-2 (Th2) subset and their cytokines have protective effects.
It has been shown that general immunosuppression (e.g. with cyclosporine) leads to a better preserved Beta-cell function (1, 2). Yet, due to the potential toxicity this approach has been discontinued in recent years.
In newer studies immunomodulators (like Anti-CD3 antibodies or a peptide from Heat shock protein -60) have been tested and have shown effects on Beta-cell-preservation (3, 4). These effects most likely result from immunomodulatory effects on Th-2/Treg activation and Th1 suppression, respectively.
Atorvastatin, which is broadly used as a lipid-lowering agent, is also a potent immunomodulator. Actually, much of its clinical benefit may be due to its property to attenuate the inflammatory component underlying atherosclerosis, metabolic syndrome and type 2 diabetes.
Statins target inflammatory responses at the level of dampening upregulation of MHC class II molecules (with an effect on T helper cell activation) or at the level of the production of inflammatory mediators such as CRP (5), but additionally by blocking the adhesion molecule LFA-1 (6). In pre-clinical trials, atorvastatin promotes a Th2 bias and reverses paralysis in central nervous system autoimmune disease (7, 8). This has already led to the initiation of a clinical trial of atorvastatin in multiple sclerosis in the USA.
The ICAM-1/LFA-1 interaction is involved in T cell migration and antigen presentation. LFA-1 interacts with the surface protein ICAM-1 on endothelial or antigen presenting cells. The latter interaction stabilizes the contact between (auto)immune helper T cells and antigen presenting cells, i.e. the immunological synapse, a well-organized site of cell-cell-interaction. Only a synapse tightly sealed by LFA-1 - ICAM-1 interactions is able to promote the activation of aggressive T helper cells of type 1 (Th1).
The Principal Investigator of the current study has done research on the interaction between ICAM-1 and its ligand for more than 10 years. Most important was his finding that the expression of ICAM-1 on immune cells is essential for the development of insulitis and diabetes in the best studied animal model of type 1 diabetes, the NOD mouse (9).
Furthermore, he discovered that soluble forms of ICAM-1, which interfere with the ICAM-1/LFA-1 interaction, can prevent diabetes onset in this animal model (Martin S, Heidenthal E, Rothe H, Schulte B, Kolb H: Soluble forms of intercellular adhesion molecule-1 intervene in the pathogenesis of autoimmune diabetes. *Diabetologia* 41: 1298-1303, 1998). For these reasons, the Principal Investigator has identified

atorvastatin as a particularly appropriate candidate for targeting immune processes involved in islet inflammation and β-cell destruction.
Further guidance come from the decision of leading experts of multiple sclerosis to do a similar trial in this debilitating immune mediated disease. It is currently believed that multiple sclerosis and type 1 diabetes share many components of etiology and immune pathogenesis.
A study in type 2 diabetic patients proved that 80 mg atorvastatin per day results in a strong decrease of high-sensitivity CRP compared to 10 mg daily or placebo. This result suggests that anti-inflammatory effects of atorvastatin might be dose-dependent. (10).
In the trial described here, the effects of 80 mg atorvastatin per day on preservation of beta-cell function in recent onset type 1 diabetes will be studied, as determined by stimulated C-peptide levels. The endpoint and study population have been chosen in accordance with the guidelines for intervention trials in newly diagnosed type 1 diabetes of the Immunology of Diabetes Society (11)

4 STUDY OBJECTIVES

The objectives of this study are:

1. To assess the effect of atorvastatin on pancreatic beta-cell function as measured by C-peptide after a liquid mixed meal stimulation in patients with newly diagnosed type 1 diabetes mellitus

2. To assess the effect on metabolic control as measured by HbA1c and insulin requirements

3. To assess the safety and tolerability of atorvastatin in the treatment of newly diagnosed type 1 diabetes patients as indicated by adverse events, changes in physical examination, ECG and vital signs and changes in laboratory parameters that are relevant to safety

4. To assess the effect on risk factors of diabetic complications as indicated by changes in serum lipids and CRP in patients with newly diagnosed type 1 diabetes

5. To assess the effect on systemic immune abnormalities in patients with type 1 diabetes mellitus as measured by effects on beta-cell autoantibodies, blood cytokines and chemokines on protein and transcriptional level

5 OVERALL STUDY DESIGN AND PROCEDURES

5.1 Study Design

This study will be conducted as a randomized, double-blind, placebo-controlled, outpatient, parallel group study in adult patients with recent onset type 1 diabetes. Patients will be randomly assigned to treatment with either atorvastatin or placebo in a one-to-one manner. The treatment phase will be 18 months for each patient. Patients who are withdrawn from the study during the first nine months of the treatment phase will be replaced. A maximum of 160 patients will complete all study procedures. The number of subjects included may be adjusted following an interim analysis as specified in section 9.9)
Starting dose will be 40 mg per day of atorvastatin or a matching number of placebo tablets. After a run-in period of 4 weeks the daily dose will be increased to 80 mg per day or matching placebo. In case of side effects (e.g. myalgia) the dose will be reduced to 40 mg per day by the discretion of the investigator.
Efficacy on beta-cell preservation will be assessed by means of two-hour mixed-meal tolerance tests at baseline and after 12 months and 18 months of treatment.
Safety data and data on metabolic control will be obtained at baseline and after 3, 6, 12 and 18 months of treatment.
Patients should continue insulin treatment throughout the study. A tight glycemic control should be aimed (Target HbA1c ≤ 6.5 %) from Visit 2 on.

5.2 Study Flow Chart

Visit	Screening	Visit 1 *	Visit 2	Visit 3	Visit 4	Visit 5	Early Discontinuation
Time	-28 to -2 days	0	3 months (±2 weeks)	6 months (± 10 days)	12 months (± 2 weeks)	18 months (± 2 weeks)	
Informed consent	X						
Inclusion/Exclusion Criteria	X						
Randomization		X					
Demographic Data	X						
Medical History and concomitant Illnesses	X						
2 h MMTT		X			X	X	
12 lead ECG	X					X	X
Vital signs	X	X	X	X	X	X	X
Body height[1], weight and BMI		X	X	X	X	X	X
Physical examination	X					X	X
Adverse Events		X	X	X	X	X	X
Concomitant Medication	X	X	X	X	X	X	X
Documentation of Insulin dose	X	X	X	X	X	X	X
Drug accountability		X	X	X	X	X	X
Hematology	X	X	X	X	X	X	X
Clinical Chemistry[1]	X	X	X	X	X	X	X
Serum Lipids	X	X	X	X	X	X	X
Coagulation	X						
Urinalysis	X						
Transcriptional studies		X	X				
Cytokines/chemokines		X	X		X	X	
Beta cell autoantibodies	X			X	X	X	
HCG[2]	X	X	X	X	X	X	X
Plasma CRP		X	X		X	X	
HbA1c	X	X		X	X	X	X
Fasting C-peptide		X[3]			X[3]	X[3]	

[1] Visit 1 only
[2]only female patients
[3] sampled during mixed meal tolerance test

* Telephone contact: Day 28/ <u>–4 days before the dose increase of the study medication</u>

5.3 Study Procedures

The patients will be selected on the basis of a screening examination. After inclusion, each patient will be required to attend the study site for five scheduled visits during the study. The patient will attend all visits in a fasted state, i.e. without food intake for at least 10 hours. Before the dose of the study medication will be increased (after 4 weeks run-in), the investigator will contact the patient by phone to make sure that no side effects occured. At visits 1, 4 and 5 the patient will be required to comply with restrictions of insulin therapy prior to the mixed meal tolerance test as outlined in Section 8.16. If a patient is acutely ill (like e.g. presence of a common cold by the scheduled time of a visit), this visit should be postponed within the time window for this visit.

The following procedures will be performed during the course of the study

5.3.1 Screening Visit

The screening visit will be performed 28 to 2 days prior to Visit 1.
Informed consent must be obtained prior to any study related activity (see section 10.3)

The following procedures will be performed (lab tests already on hand and not older than 28 days at time ofvisit 1 will not be repeated):

Check of inclusion and exclusion criteria (see sections 6.3 and 6.4)
Demographic data (see section 8.1)
Medical history and concomitant illnesses (see section 8.2)
Concomitant medication including insulin dose (see section 8.3)
Vital signs (see section 8.5)
Physical examination (see section 8.6)
12-lead ECG (see section 8.7)
Safety laboratory including urinalysis and coagulation (see section 8.8)
Serum Lipids (see section 8.9)
HCG (in women only) (see section 8.10)
Beta-cell autoantibodies (see section 8.12)

5.3.2 Visit 1

Visit 1 should be performed 28 days to two days after screening visit. Visit 1 should not be scheduled later than 3 months and not earlier than two weeks after the start of the insulin treatment of the patient.

The following procedures will be performed:

Randomization (see section 7.5)
Questioning for Adverse events (see section 8.17)
Concomitant medication including insulin dose (see section 8.3)

Body weight, height and BMI (see section 8.4)
Vital signs (see section 8.5)
Safety laboratory (see section 8.8)
Serum Lipids (see section 8.9)
HCG (in women only) (see section 8.10)
HbA1c and fasting C-peptide (see section 8.11)
Plasma CRP (see section 8.13)
Serum cytokines and chemokines (see section 8.14)
Transcriptional analysis (in a subset of 50 patients) (see section 8.15)
Two-hour mixed-meal tolerance test (see section 8.16)
Dispensing of study medication (see section 7.3)

5.3.2.1: Telephone contact

A telephone contact will be performed 4 weeks after the start of the run-in period (Day 28/-4 days), before the dose of the study medication will be increased from 40 to 80 mgs daily.

The following questions will be asked:

- **Adverse events in general ("Have you experienced any problems since your last visit/contact?")**
- **Muscle related adverse events in particular; the investigator should pose the following three questions:**

1. **Did you suffer of any muscle pain during the last four weeks?**
2. **Did any muscle ache occur during the last four weeks that cannot be explained by unusual strenuous physical activity?**
3. **Did you experience any weakness of muscles during the last four weeks?**

5.3.3 Visit 2

Visit 2 should be performed 3 months (± 2 weeks) after Visit 1.

The following procedures will be performed:

Questioning for Adverse events (see section 8.17)
Concomitant medication including insulin dose (see section 8.3)
Body weight and BMI (see section 8.4)
Vital signs (see section 8.5)
Safety laboratory (see section 8.8)
Serum Lipids (see section 8.9)
HCG (in women only) (see section 8.10)
Plasma CRP (see section 8.13)
Serum cytokines and chemokines (see section 8.14)
Transcriptional analysis (in a subset of 50 patients) (see section 8.15)
Collection of used, unused and partly used study medication and dispensing of study medication (see section 7.3)

5.3.4 Visit 3

Visit 3 should be performed 6 months (± 10 days) after Visit 1.

The following procedures will be performed:

Questioning for Adverse events (see section 8.17)
Concomitant medication including insulin dose (see section 8.3)
Body weight and BMI (see section 8.4)
Vital signs (see section 8.5)
Safety laboratory (see section 8.8)
Serum Lipids (see section 8.9)
HCG (in women only) (see section 8.10)
Beta-cell autoantibodies (see section 8.12)
Collection of used, unused and partly used study medication and dispensing of study medication (see section 7.3)
HbA1c (see section 8.11)

5.3.5 Visit 4

Visit 4 should be performed 12 months (± 2 weeks) after Visit 1.

The following procedures will be performed:

Questioning for Adverse events (see section 8.17)
Concomitant medication including insulin dose (see section 8.3)
Body weight and BMI (see section 8.4)
Vital signs (see section 8.5)
Safety laboratory (see section 8.8)
Serum Lipids (see section 8.9)
HCG (in women only) (see section 8.10)
HbA1c and fasting C-peptide (see section 8.11)
Beta-cell autoantibodies (see section 8.12)
Plasma CRP (see section 8.13)
Serum cytokines and chemokines (see section 8.14)
Two-hour mixed-meal tolerance test (see section 8.16)
Collection of used, unused and partly used study medication and dispensing of study medication (see section 7.3)

5.3.6 Visit 5

Visit 5 should be performed 18 months (± 2 weeks) after Visit 1.

The following procedures will be performed:

Questioning for Adverse events (see section 8.17)
Concomitant medication including insulin dose (see section 8.3)
Body weight and BMI (see section 8.4)
Vital signs (see section 8.5)

Physical examination (see section 8.6)
12-lead ECG (see section 8.7)
Safety laboratory (see section 8.8)
Serum Lipids (see section 8.9)
HCG (in women only) (see section 8.10)
HbA1c and fasting C-peptide (see section 8.11)
Beta-cell autoantibodies (see section 8.12)
Plasma CRP (see section 8.13)
Serum cytokines and chemokines (see section 8.14)
Two-hour mixed-meal tolerance test (see section 8.16)
Collection of all used, unused and partly used study medication (see section 7.3)

5.3.7 Early Discontinuation Visit

If the patient is prematurely discontinued from further study participation he/she will be invited to attend an early discontinuation visit. If the reason for discontinuation was an adverse event that is still present by the time of the early discontinuation visit, the adverse event will be followed up until it is resolved or stabilized.

The following procedures will be performed:

Questioning for Adverse events (see section 8.17)
Concomitant medication including insulin dose (see section 8.3)
Body weight (see section 8.4)
Vital signs (see section 8.5)
Physical examination (see section 8.6)
12-lead ECG (see section 8.7)
Safety laboratory (see section 8.8)
HCG (in women only) (see section 8.10)
HbA1c (see section 8.11)
Collection of all used, unused and partly used study medication (see section 7.3)

6 SELECTION OF STUDY POPULATION

The selection of study population should follow the guidelines by the International Immunology of Diabetes Society (11).

6.1 Primary Diagnosis

Male or female patients with the diagnosis of type 1 diabetes mellitus based on the criteria of the American Diabetes Association (12) no more than 3 months from start of insulin therapy.

6.2 Number of Subjects

It is aimed that a maximum of 160 patients will complete all study procedures. Patients will be randomized one-to-one to the active treatment or the placebo group. The number of patients may be adjusted following the interim analysis (see section 9.9).

Patients must meet all of the inclusion criteria and not meet any of the exclusion criteria before the first administration of study drug to be eligible for the study.

6.3 Inclusion Criteria

1. Insulin treated patients with a newly diagnosed type 1 diabetes mellitus as defined by the ADA criteria at least two weeks but not later than 3 months after start of insulin treatment
2. Age18 to 39 years, inclusive
3. Male patient or female patient using adequate contraceptive methods (oral contraceptives, intrauterine device, sterilization or consistent use of barrier methods like diaphragm and/or condoms in combination with spermicides) throughout the study
4. Tested positive for at least one of the three islet autoantibodies GAD65, IA2 or ICA

6.4 Exclusion Criteria

1. History of a malignancy
2. Presence of a clinically significant hepatic or renal disease, as indicated, but not limited to a serum creatinine elevated more than ten percent above the upper limit of normal, elevation of AST or ALT more than 3 times the upper limit of normal

3. Any other acute or chronic condition that may affect the patient's response to treatment or might be associated with an increased risk for the patient to participate, as judged by the investigator
4. Current use of anti-inflammatory or immunmodulatory drugs, antihypertensive, lipid-lowering, or antidiabetic drugs other than insulin
5. Pregnant or nursing women or women intending to become pregnant during the course of the study
6. Patients with any significant diseases or conditions, including psychiatric disorders and drug abuse, that in the opinion of the investigator, are likely to affect the patient's response to treatment or his/her ability to comply with study procedures
7. Known or suspected allergy to atorvastatin or any component of the trial product
8. Known myopathy, myalgia or myositis with a serum CPK above 3 times the upper limit of normal
9. A serum-CPK above 5 times the upper limit of normal
10. Patients who have received any investigational drug within 3 months prior to Visit 1
11. Patients who had a severe blood loss (≥ 400 ml, e.g. blood donation) within 2 months prior to Visit 1
12. Patients have any significant laboratory abnormality at time of screening, such hemoglobin < 11 mg/dL, or any other clinically significant abnormal laboratory test result, as judged by the investigator
13. A serum LDL-cholesterol above 150 mg/dL at time of screening
14. Unwillingness to comply with the study procedures, as judged by the investigator

6.5 Withdrawal Criteria

During the study, each subject may withdraw from further participation at any time and without giving reasons. However, adequate efforts should be made to determine the reasons for discontinuing. If a patient is lost to follow-up, adequate effort should be made to determine the whereabouts and medical status of that patient, and to recover the study medication.

Patients may be discontinued early for any of the following reasons:

- An adverse event too severe to allow continuation
- Gross patient non-compliance (e.g. if compliance of drug intake is less than 80% on two consecutive visits)
- A significant protocol violation as determined by the Study Committee
- Elevation of ALT or AST of more than 3 times the upper limit of normal
- Elevation of CPK more than 10 times the upper limit of normal, or presumed or assured diagnosis of a rhabdomyolysis
- Elevation of serum-creatinine more than 2 mg/dL
- Pregnancy or nursing occurring during the trial
- Intake of a HMG-CoA reductase inhibitor (other than the study medication) during the course of the study

If a patient is withdrawn from the study the patient should be invited to attend an Early Discontinuation Visit, the reasons for removal from the study and the date of last use of study medication should be recorded.
Patients discontinuing because of an adverse event must continue to maintain contact until the event resolves or is stable, and the information must be documented by the investigator.
Patients who did not receive any medication because of withdrawal prior to the administration of the first dose will not be assigned a patient number, and data obtained up to that point will not be entered in the study database.
Patients withdrawn from the study during the first nine months after the first intake of study drug will be replaced.

7 TREATMENTS TO BE ADMINISTERED

Atorvastatin (or matching placebo) will be supplied as 40 mg tablets for oral administration. The patients will take study medication in the evening on a daily base. The daily dose of atorvastatin or (matching number of Placebo tablets) will be 40 mg during the first four weeks of treatment and 80 mg from the fifth week till the end of the study. In case of side effects (e.g. myalgia) the dose may be reduced to 40 mg/day by the discretion of the investigator.

7.1 Identity of Investigational Product

To achieve blinding of the study medication to patients and investigators, atorvastatin and placebo will be indistinguishable from each other on the basis of all directly observable characteristics such as shape, size, appearance, weight, taste, odor, finish and dissolution characteristics.

7.2 Labeling and Packaging of the Investigational Product

Packaging, labeling and shipping of the investigational product will be performed by Profil GmbH in accordance with Profil's applicable standard operating procedures Study medication will be provided in containers which will be closed with a child-resistant closure. There will be a label bearing information that will meet the applicable regulatory requirements. The study medication will be supplied with a certificate of analysis.

7.3 Drug Accountability

It is the responsibility of each investigator to ensure that all study drug received at the site will be inventoried and accounted for throughout the study and the result recorded in the drug accountability form maintained in the investigator's study file. Patients must be instructed to return all original containers, whether empty or containing study drug. Study drug returned by patients will be stored and disposed of according to the manufacturer's instructions. Contents of the study drug containers must not be combined. The drug accountability will be verified by the site monitor during on-site monitoring visits. Study drug will be stored under appropriate environmental conditions.
The study drug will not be supplied to any person other than investigators, designated staff and the patients participating in the study. Study drug may not be relabeled or reassigned for use by other subjects except under special circumstances approved by the responsible Study Coordinator at Profil GmbH.
The investigators will retain and store all original containers returned by subjects, until these containers are inventoried by Profil. The investigators agree to return all

original containers at the end of the study, whether empty or containing study drug, to Profil as instructed by the site monitor. The investigators agree neither to dispense the study drug from, nor store it at, any site other than the study sites agreed upon with Profil.
Profil will ensure proper disposition of original containers whether empty or full with returned or unused study drug. Appropriate documentation will be maintained. Permission may be granted for local disposition, with supporting documentation.

7.4 Blinding

Blinded treatment will be used to reduce potential bias during data collection and evaluation of clinical endpoints.
The investigators will have code break envelops to be opened upon specific instructions by Profil's Study Coordinator or in case of life-threatening situation which is supposed to be related to the study drug or the study drug might interfere with the patient's further medical care. The study coordinator/monitors should be informed within 24 hours about the code break. Individual code breaks by an investigator will normally result in withdrawal of the subject from the trial. The date, time, and reason for the unblinding must be documented on the appropriate page of the CRF and in the source document.

7.5 Randomization

Randomization will be used to avoid bias in the assignment of subjects to treatment, to increase the likelihood that known and unknown patient attributes (e.g., demographics and baseline characteristics) are evenly balanced across treatment groups, and to enhance the validity of statistical comparisons across treatment groups.
Randomization will be performed before or at Visit 1, after the results of all screening assessments are available. Each patient will be randomly assigned to one of the two possible treatment groups.
Each study site will be assigned a block of randomization numbers. The study medication will be supplied in containers with the randomization number specified on the label.
For each randomization an investigator will contact Profil, where an unblinded person who is otherwise not involved in study procedures will assign a randomization number by means of a randomization list.

7.6 Treatment Compliance

The investigator or designated study personnel will maintain a log of all study drug dispensed and returned. Drug supplies for each subject will be inventoried and accounted for throughout the trial.
Compliance will be determined by a capsule count at each study visit. If compliance is < 80 % at two consecutive visits the subject must be withdrawn.

8 STUDY ASSESSMENTS

8.1 Demographic Data

The following information will be obtained and documented at Screening visit:

- Date of birth
- Gender
- Family history of diabetes for first degree relatives
- Smoking History
- Ethnic Origin

8.2 Medical History, including Concomitant Illnesses

All relevant medical conditions and illnesses in the past and concomitant illnesses and conditions present at study entry will be documented at Screening Visit, including the date of diagnosis of type 1 diabetes and the presence or absence of ketoacidosis.

8.3 Concomitant Medication, including insulin requirements

Details on Concomitant Medication will be obtained at Screening Visit and all subsequent visits, including start date and stop date, frequency, and route of application.
Insulin treatment should be continued throughout the study with a recommended treatment goal of HbA1c below 6.5% from Visit 2 on.
However, missing this treatment goal will not be a reason for discontinuing a patient from further participation.
It is of uttermost importance, that the start date of insulin therapy is obtained and the current insulin dose is thoroughly documented at each visit.
The current insulin dose (the daily dose of all sorts of insulin) will be summed up and the weight-corrected insulin requirements will be calculated (expressed as IU per kg).

8.4 Body weight, Height and BMI

Height, without shoes (m) will be measured at Visit 1.
Weight, without shoes and overcoat (kg) will be recorded at Visit 1 and all subsequent visits.
Body Mass Index will be calculated based on Body weight and height (kg/m^2).

8.5 Vital Signs

Heart rate (beats per minute), diastolic and systolic blood pressure (mmHg), in a supine position after5 minutes rest, will be measured at Screening Visit and all subsequent visits.

8.6 Physical Examination

A complete physical examination will be performed by an investigator at Screening Visit, , Visit 5 and, if applicable, Early Discontinuation Visit. Any abnormality will be recorded and described at Screening Visit and any significant changes will be recorded at subsequent visits.

8.7 12-Lead ECG

A standard 12-lead ECG will be recorded at Screening Visit, Visit 5 and, if applicable, Early Discontinuation Visit. Any abnormality will be recorded and described at Screening visit and any significant changes will be recorded at subsequent visits.

8.8 Safety Laboratory

8.8.1 Hematology

The following parameters will be determined at Screening Visit, Visits 1 to 5 and, if applicable, Early Discontinuation Visit:
Leukocytes, erythrocytes, hemoglobin, hematocrit, MCV, MCH, MCHC, platelets.Differential blood cell count will be determined at Screening Visit and Visit 5 (or Early Discontinuation Visit)

8.8.2 Clinical chemistry

The following parameters will be determined at Screening Visit and all subsequent Visits:
AST, ALT, CPK, creatinine, sodium, potassium, calcium, chloride, total protein, GGT, AP, LDH.

8.8.3 Urinalysis

A urine sample will be obtained at Screening Visit. The urinalysis will comprise the following parameters:
Blood, pH, protein, glucose, ketones, sediment.

8.8.4 Coagulation

A blood sample for coagulation parameters (PTT and INR) will be obtained at Screening Visit.

8.9 Serum Lipids

Serum Lipids will be tested at Screening Visit, Visits 1 to 5 and , if applicable, at Early Discontinuation Visit.
Serum samples will be obtained after fasting for at least 10 hours. The following parameters will be tested:
Total cholesterol, HDL-cholesterol, LDL-cholesterol, and triglyerides.
The tests will be performed in a central laboratory for all study sites.
From Visit 1 to 5 the investigators will be blinded for the results of the serum lipid measurements to ensure that no conclusions on the treatment group can be drawn.

8.10 Pregnancy Test

In women, a pregnancy test will be performed at screening and all subsequent visits and at any time during the study, when a pregnancy is assumed.

8.11 HbA1c and Fasting C-peptide

Samples for HbA1c will be obtained at Screening Visit and at Visits 1, 3, 4 and 5.
Fasting C-peptide samples will be obtained during mixed meal tolerance tests at Visits 1, 4 and 5.

8.12 Beta-cell Autoantibodies

The following Autoantibodies will be determined at Screening Visit and at Visits 3, 4, and 5:

- GAD65
- IA2
- ICA

8.13 Plasma CRP

Plasma-CRP will be tested at Visits 1, 2, 4 and 5.

8.14 Serum Cytokines and Serum Chemokines

Blood samples for assays on immune mediators will be performed at Visits 1, 2, 4 and 5. Tests will be done for acute phase proteins (CRP, Fibrinogen), soluble adhesion molecule (sICAM-1), cytokines (IL-4, IL-5, IL-6, IL-10, IL-12, IL-13, IL-18 and IFN-gamma) and chemokines (MIP-1 alpha, MCP-1, IP-10, Eotaxin, MIP-2). This selection of parameters may be fractionally changed, if needed.
An additional 1ml serum and 0.5 ml plasma will be collected at these visits for possible additional markers on protein or transcriptional level.

8.15 Transcriptional Analysis

At Visits 1 and 2, Serum and Plasma in a subset of 50 patients will be sampled for analyses of expression of immune mediators on transcriptional level. A laboratory manual of the procedures and details of the tests will be provided prior to the start of the study.

8.16 Two Hour Mixed Meal Tolerance Test

A mixed-meal tolerance test will be performed at Visit 1, Visit 4 and Visit 5.
Patients will refrain from alcohol intake and unaccustomed strenuous physical activity for 48 hours prior to the test.
Prior to the test a capillary blood glucose measurement will be done and the test will only be performed, if fasting blood glucose is ≥ 4 mmol/l (72 mg/dl) and ≤ 11.1 mmol/l (200 mg/dl). If the fasting blood glucose is outside of this range, the test may be rescheduled once within the time window for this visit.
The test will be performed in the morning between 7 and 10 a.m.. The patient should take no short-acting insulin at least 6 hours prior to the test. Patients on continuous subcutaneous insulin infusion therapy should continue with the normal basal rate, but add no boluses at least 6 hours prior to the test. Patient will be fasted and will have had no food or drink (with the exception of water) and no smoking since 10 p.m. the preceding day. An I.V. line will be applied (EMLA cream may be used). Two blood samples will be drawn 5 min. and immediately before the liquid meal is taken (formal "time zero"). The patient will drink the standardized liquid meal: Boost HP (High Protein)®(Meade Johnson) in a dose of 6 ml per kg body weight with a maximum of 360 ml. The test meal should be ingested within 5 minutes. A blood sample will be drawn 90 min after the end of the ingestion of BoostHP®. (Please note that time runs from the completion of ingestion). After the test will be completed, the patients may eat and get insulin as prescribed by the investigator.
Serum glucose and C-peptide (RIA) will be analyzed in a central laboratory.
The following parameter will be calculated for efficacy analysis:
Total area under the C-peptide curve, incremental area under the C-peptide curve after test meal.

8.17 Adverse Events

Each patient must be carefully monitored for adverse events – this includes clinical laboratory test variables. At each evaluation, the investigator will determine whether any adverse events have occurred. The patient will be questioned in a general manner and no specific symptoms will be suggested during the questioning. If any adverse events have occurred, they will be recorded on the adverse event pages of the CRF. If known, the diagnosis should be recorded in preference to the listing of individual signs and symptoms. An assessment must be made of the seriousness, intensity, and relationship to the administration of the study medication.

8.17.1 Definition of Adverse Events

Adverse Event
Any undesirable event occurring to a patient during a clinical trial, whether considered related to the investigational product or not.

Worsening of the Condition Being Investigated or of Concurrent Conditions
The worsening or exacerbation of any concurrent illness or condition will be considered an adverse event. If the symptoms of the condition being treated with study medication worsen after the start of administration of drug, these symptoms will be considered adverse events if, in the investigator's opinion, their occurrence or severity is outside of what is expected.

Relationship to investigational product:
The assessment of the relationship of an adverse event to the administration of study drug (*none, unlikely (remote), possible, probable, not assessable*) is a clinical decision based on all available information at the time of the completion of the case report form.
None - includes: (1) the existence of a clear alternative explanation (e.g. mechanical bleeding at surgical site); or (2) non-plausibility (e.g., the patient is struck by an automobile at least where there is no indication that the drug caused disorientation that may have led to the event; cancer developing a few days after drug administration).
Unlikely (remote) - a clinical event, including a laboratory abnormality, with an improbable time sequence to drug administration and in which other drugs, chemicals or underlying disease provide plausible explanation.
Possible - a clinical event, including a laboratory abnormality, with a reasonable time sequence to administration of the drug, which could also be explained by concurrent disease* or other drugs or chemicals. Information on drug withdrawal may be lacking or unclear.
Probable - a clinical event including a laboratory abnormality, with a reasonable time sequence to administration of the drug, unlikely to be attributed to concurrent disease* or other drugs or chemicals, and which follows a clinically reasonable response on withdrawal (dechallenge).
Not assessable - a report of an adverse event which cannot be judged because information is insufficient or contradictory, and which cannot be supplemented or verified.
* Concurrent disease includes concomitant, intercurrent and underlying disease/condition. Concomitant disease - any other illness the subject may have at

the time of entering the clinical trial. Intercurrent disease - any other illness the subject may develop during the clinical trial. Underlying disease - the illness which is the indication for study drug therapy.

Factors to be considered include:
- *The temporal sequence from drug administration* (The event should occur after the drug is given. The length of time from drug exposure to event should be evaluated in the clinical context of the event.)
- *Recovery on discontinuation (dechallenge), recurrence on reintroduction (rechallenge)* (Subject's response after drug discontinuation (dechallenge) or subjects response after drug re-introduction (rechallenge) should be considered in the view of the usual clinical course of the event in question.)
- *Underlying, concomitant, intercurrent diseases* (Each report should be evaluated in the context of the natural history and course of the disease being treated and any other disease the subject may have.)
- *Concomitant medication or treatment* (The other drugs the subject is taking or the treatment the subject receives should be examined to determine whether any of them may be recognized to cause the event in question.)
- *Known response pattern for this class of drug* (Clinical/preclinical.)
- *Exposure to physical and/or mental stresses* (The exposure to stress might induce adverse changes in the recipient and provide a logical and better explanation for the event.)
- *Pharmacology and pharmacokinetics of the test drug* (The pharmacokinetic properties (absorption, distribution, metabolism and excretion) of the test drug (s) the subject is taking, coupled with the individual subject 's pharmacodynamics should be considered.)

Intensity (Severity) of the Event
In addition to assessing the connection with administration of the investigational drug, the intensity (severity) of each adverse event must be assessed. The following classification should be used:
- *Mild* usually transient in nature and generally not interfering with normal activities
- *Moderate* sufficiently discomforting to interfere with normal activities.
- *Severe* prevents normal activities.

8.17.2 Definition of Serious Adverse Events

Serious adverse event
A serious adverse event is defined as any untoward medical occurrence that:
- results in death
- is life- threatening (note: the term life threatening indicates that the patient was at immediate risk of death)
- results in inpatient hospitalization/ prolongation of hospitalization
- results in persistent or significant disability/incapacity
- results in a congenital anomaly or birth defect.

Important medical events that may not result in death, be life-threatening, or require hospitalization may be considered serious adverse drug events when, based upon appropriate medical judgment, they may jeopardize the subject and may require medical or surgical intervention to prevent one of the outcomes listed in this definition. Examples of such medical events include allergic bronchospasm requiring

intensive treatment in an emergency room or at home, blood dycrasias or convulsions that do not result in inpatient hospitalization, or the development of drug dependency or drug abuse.
Life-threatening means that the patient was, in the view of the investigator, at immediate risk of death from the reaction as it occurred. This does not include an adverse event that, had it occurred in a more serious form, might have caused death.
Disability means a substantial disruption of a person's ability to conduct normal life's functions.

8.17.3 Change of Laboratory Parameters

Laboratory Test Variable as an Adverse Event
A laboratory test variable that is abnormal or has changed significantly since baseline should be recorded as an adverse event if it causes the patient to be discontinued from the study, there are clinical manifestations, or treatment is required.

8.17.4 Documentation and Reporting of Adverse Events

All adverse events occurring during the trial and follow-up period must be fully recorded in the patient's CRF.
The investigator or staff member must report all adverse events encountered during the clinical study, whether or not the event is considered drug-related.
The signs and symptoms of the individual adverse event must be described in detail: date of onset and end, intensity, relationship to the test drug, action taken and outcome.
Documentation must be supported by an entry in the patient's files.
The following data should be documented for each adverse event:

- Study code number.
- Patient identification number.
- Description of the symptom/event.
- Classification of "serious" or "not serious".
- Intensity.
- Date of first and last occurrence.
- Frequency (once, occasionally, frequently, permanently).
- Treatment required (no treatment necessary, treatment with prescription only drug(s), outpatient treatment (with dates recorded), inpatient treatment (hospitalization), prolonged hospitalization (with dates recorded).
- Causal relationship to investigational product(s) (according to CPMP guideline III/3445/91).
- Outcome of event (unknown, recovered, not yet recovered, sequelae with disability/incapacity, death (with date and cause recorded).

Any adverse event (serious or otherwise), that could affect adversely the safety of patients or the conduct of the trial should be reported to Profil by telephone and/or fax within 24 hours using the adverse event expedited reporting form provided. Profil will inform Pfizer Deutschland GmbH within 24 hours after the event has been reported.

The information must comprise at least the following data:

- Name, address and telephone number of the reporting investigator.
- Investigational product.
- Study code.
- Patient identification number, initials, sex and date of birth.
- Description of the adverse event, measures taken and outcome.
- Preliminary classification of causal relationship by the investigator according to CPMP guideline III/3445/91

This information can be transmitted via telephone or fax.

On behalf of Pfizer Deutschland GmbH, Profil will promptly notify all relevant investigator(s)/institution(s) and the regulatory authority(ies) of findings that could affect adversely the safety of patients, impact on the conduct of the trial, or alter the ethics committee approval/favorable opinion of the trial. In addition, Profil will expedite the reporting to all concerned investigator(s)/institutions(s), to the ethics committees, where required, and to the regulatory authority(ies) of all adverse events that are both serious and unexpected.

9 STATISTICAL EVALUATION

9.1 Determination of sample size

For the primary endpoint the calculation of sample size is based on data on levels and variation taken from a recent study at the German Diabetes Clinic. After 12 months of intense insulin treatment, stimulated C-peptide was 0.37 ± 0.053 nmol/l (mean ± standard deviation) With a sample size of 80 patients per group a 20% difference in stimulated C-peptide would be significant with a p-value < 0.01.

9.2 Statistical and Analytical Plan

The statistical evaluation will be performed by using validated software. All data will be listed and trial summary tables will be provided. Descriptive summary statistics will be presented by group (Verum, Placebo). Standard statistical tests will be applied. A detailed plan for statistical analysis will be amended.

9.3 Demographics and Baseline Medical Characteristics

Summary statistics will be presented. Frequency tables for qualitative data will be provided. The two groups will be compared for balance in age by means of an unpaired t-test.

9.4 Definition of Study Populations for Analysis

All patients who received at least one dose of study treatment will be included in the analysis of safety.
All patients who complete the study, will be included in the analysis of immune response and efficacy. The immune results of patients who drop out before completion of the study will be analyzed in a descriptive manner. If the study should be prematurely stopped after an interim analysis (see section 9.9), the study population for clinical efficacy will be defined as all patients who completed Visit 5 after 12 Months of treatment.

9.5 Handling of Missing and Incomplete Data

Missing or incomplete data will be reported as deviations from the protocol.

9.6 Safety Evaluation

All randomized patients who take at least one dose of study medication will be included in the safety analysis. The incidence of adverse events, drug-related adverse events and premature discontinuations will be summarized descriptively for each treatment group. The incidence of treatment emergent ECG-findings and of abnormally high and low laboratory values will be presented by treatment group. Descriptive statistics for changes from baseline in vital signs and laboratory values will be presented by treatment group.

9.7 Evaluation of Immune Response

Patients will be included in the evaluation of immune response as outlined in section 9.4.
If appropriate, the results may be stratified for age, status of autoimmune antibodies and C-peptide levels at the entry into the study.

9.8 Evaluation of Clinical Efficacy

Patients will be included in the evaluation of clinical efficacy as outlined in section 9.4. If appropriate, the results may be stratified for time from diagnosis to visit 1, gender, age, status of autoimmune antibodies and C-peptide levels at the entry into the study.
The concentration-time courses of basal C-peptide secretion will be summarized separated by treatment. Standard parameters of descriptive statistics will be calculated for each of the sampling points and for the area under the C-peptide concentration curves. Means at any time will only be calculated, if at least 2/3 of the individual data were measured and were above the limit of quantification (LOQ). For the calculation of the mean value a data point below LOQ will be substituted by half of this limit. Where values below LOQ are included in the calculation of mean values, these means will be marked in the relevant summary table.
C-peptide values will be compared for the two treatment groups using standard statistical tests that will be specified in a statistical plan that will be amended.
Area under the C-peptide curve of the MMTT will be analyzed as mentioned above. Other efficacy parameters like C-peptide at different sampling times, maximum C-peptide concentration or incremental area under the C-peptide curve may be calculated where appropriate.
Statistical analyses of weight corrected Insulin-doses, HbA1c, Serum-lipids and Serum-CRP levels will be done accordingly.

9.9 Interim Analyses

Informal interim safety analyses will be performed at 6-monthly intervals during safety discussions between the Study Committee and the independent external Safety Review Board. Decisions will be made on the discretion of the investigators and the

Safety Review Board. Any changes to the study as a result of the data review will be made as amendments to the protocol.
In addition an interim analysis for clinical efficacy and immunology parameters will be performed after 40 percent of both treatment groups have completed Visit 4 (12 months of treatment). For this analysis the statistical methods described above will be applied.
At this interim analysis the determination of sample size may be adjusted based on the clinical efficacy data so far obtained. If a clinically relevant and statistically significant difference for the primary efficacy endpoint can be assessed at this interim analysis, the study can be prematurely stopped by the discretion of the Study Committee and the Safety Review Board.

10 ETHICS

10.1 Independent Ethics Committee

Prior to initiation of the study the protocol, any amendments, informed consent form, the patient information sheet and any other written information to be provided to the subject, details on the patient recruitment procedures and any other relevant study documentation required will be submitted to the responsible Independent Ethics Committee. Written approval of the study must be obtained prior to commencement of the trial.
The investigator will report promptly to the Ethics Committee, any new information that may adversely affect the safety of patients or the conduct of the trial. Similarly, the investigator will submit written summaries of the trial status to the Ethics Committee annually, or more frequently, if requested by the Ethics Committee. Upon completion of the trial, the investigator will provide the Ethics Committee with a brief report of the outcome of the trial, if required.

10.2 Ethical Conduct of the Study

The trial will be conducted in accordance with the Declaration of Helsinki in its revised version (Edinburgh, 2000) and adhere to the ICH GCP guideline as well as to the demands of applicable regulatory requirements.

10.3 Patient Information and Consent

The investigator is responsible for ensuring that no patient is subject to any study-related activity before that patient has given informed consent.
A voluntary, signed and dated informed consent form must be obtained from the patient after the investigator has given the patient oral and written information about the trial. The investigator will inform the patient of the aims, methods, anticipated benefits and potential hazards of the study including any discomfort it may entail. The patient must be given every opportunity to clarify any points he/she does not understand and if necessary ask for more information. At the end of the interview the patient may be given time to reflect, if this is required, or if the patient requests more time.
After completion, informed consent forms will be kept and archived by the investigator in the investigator’s study file.
It should be emphasized that the patient is at liberty to withdraw their consent at any time, without loss of benefits to which the patient is otherwise entitled. Patients who refuse to give, or withdraw, written informed consent may not be included or continued in this study.

11 DOCUMENTATION, RECORD ACCESS AND ARCHIVING

11.1 Documentation of Patients' Participation

For all patients who give informed consent, regardless of whether they receive any investigational product, the investigator must record patient identification data in the "Patient Identification List" (full name, initials, date of birth, patient identification code). The patient identification list must allow for the definite identification of any patient that takes part in the study. The investigator must keep the list of patient identification codes for a period of at least 15 years after completion of the study within the investigator’s study file.
A statement acknowledging the participation of a patient in this clinical trial must be documented in the patient's medical file/notes and, where the study physician is not the primary care physician, it is recommended that the patient’s primary care physician is informed of his/her patient’s participation in this clinical study, provided the patient gave his/her consent.

11.2 Documentation of Essential Documents/Supplements at Study Site During the Trial

At the beginning of the study, an Investigator’s Study File will be established at every study site. The investigator/institution is responsible for maintaining the trial documents as specified in the Guideline for GCP (CPMP/ICH/135/95) and as required by the applicable regulatory requirement(s). The investigator/institution must take measures to prevent accidental or premature destruction of these documents.

11.3 Patients' Records

The investigator shall permit Profil and regulatory agency employees to enter and inspect any site where the drug or records pertaining to the drug are held, and to inspect and copy all records relating to an investigation, including patient records. Completed CRFs must be made available for review by Profil’s Clinical Monitor and the regulatory agencies. To ensure the accuracy of data submitted, it is mandatory that representatives of Profil and of the regulatory agencies have access to source documents (i.e., patient medical records, charts, laboratory reports, etc.). Patient confidentiality will be protected at all times. A CRF will only be considered completed when each CRF section has been reviewed and signed by the investigator indicating his/her assurance of the accuracy of all recorded data. It is expected that the investigator and his/her staff will cooperate with the Study Monitor and provide any missing data in a timely manner.

11.4 Case Report Forms

The CRF, as well as the protocol, are confidential.

- The CRFs will be designed and supplied by Profil. The original is to be returned to Profil.
- All CRFs are to be completely filled out by the examining personnel and reviewed and signed by the investigator(s).
- All CRFs are to be completed in a neat, legible manner to ensure accurate interpretation of data. A black pen is preferred to ensure clarity of any reproduced copies of all CRFs.
- Any change or corrections made on the CRFs must be dated and initialed by the one making the changes. In such cases, the best procedure is to cross out original entry. **DO NOT ERASE, OVERWRITE, OR USE LIQUID PAPER ON ORIGINAL ENTRY**
- It is each investigator's responsibility to ensure that all DISCONTINUED orders or changes in study or other medications entered on the subject's CRF correspond to the entries on the subject's medical records.
- The CRFs for any subject leaving the study should be completed at the time medication is terminated.
- CRFs should accurately reflect data contained in patients' records (i.e., source documents).

11.5 Archiving

The originals of the completed CRFs will be returned to Profil at the end of the study. No-carbon-required copies of CRFs and documentation of informed consent will be retained by the investigator with the patient's notes.
Copies of all study documents will be retained by Profil for at least 15 years following the end of the study.

12 QUALITY ASSURANCE AND QUALITY CONTROL

Steps to be taken to assure the accuracy and reliability of data include the selection of qualified investigators and appropriate study sites, review of protocol procedures with the investigator and associated personnel prior to the study, periodic monitoring visits by Profil. The CRFs will be reviewed for accuracy and completeness by Profil during on-site monitoring visits and after their return, and any discrepancies will be resolved with the investigator or designees, as appropriate. The data will be entered into the clinical trial data base and verified for accuracy.

12.1 Monitoring

Monitors will establish and maintain regular contact between the investigator and Profil GmbH. All clinical work conducted under this protocol is subject to authorities

and regulatory regulations/guidelines including an inspection by Profil or Health Authorities at any time. The clinical investigator must agree to the inspection of study related records by government regulatory agencies or Profil.

It is the responsibility of the investigator to ensure that the study is conducted in accordance with the protocol, ICH GCP and the Declaration of Helsinki and that valid data are entered into the CRFs. To achieve this objective, the Study Monitor's duties are to aid the investigator in the maintenance of complete, legible, well organized and easily retrievable data. In addition, the Study Monitor will explain, interpret and ensure the investigator's understanding of all applicable regulations concerning the clinical evaluation of the study medication and ensure an understanding of the protocol, reporting responsibilities and the validity of the data.

Monitors will evaluate the competence of each study site relating to facilities, technical equipment or medical staff. During the study, monitors will check that written informed consent has been obtained from all patients correctly and that data are recorded correctly and completely. Monitors are also entitled to compare entries in CRFs with corresponding source data and to inform the investigator of any errors or omissions. Monitors will also control adherence to the protocol at the investigator site. They will arrange for the supply of investigational product and ensure appropriate storage conditions are maintained.
Monitoring visits will be made to the sites as frequent as appropriate whilst patients are ongoing in the study. The monitor will make written reports on each occasion contact with an investigator is made.
During monitoring visits, entries in the CRFs will be compared with the original source documents (source data verification). For the following items, this check will be 100%:

- patient identification number
- patient consent obtained
- patient eligibility criteria (inclusion and exclusion criteria)
- efficacy variables
- medical record of adverse events.

For all other items, at least 20% of the data will be checked.

12.2 Data Management/Coding

Data generated within this clinical trial will be handled according to the relevant SOPs of Profil or the SOPs of Profil's sub-contractor, where applicable. Double data entry into the computer will be done by two independent persons.

12.3 Audit and Supervision of the Study

Investigator sites, the study database and study documentation may be subject to Quality Assurance audit during the course of the study by Profil. In addition, inspections may be conducted by regulatory bodies at their discretion.

13 ADMINISTRATIVE PROCEDURES

13.1 Amendments to the Protocol

Modifications of the signed protocol are only possible by approved protocol amendments and with the agreement of all responsible persons. The procedure for approval of a protocol amendment is identical to that for approval of the protocol. The Ethics Committee must be informed of all protocol amendments and should be asked for its opinion as to whether a re-evaluation of the ethical aspects of the trial is necessary.
No deviation from, or change to the protocol, may be implemented without agreement by the Study Committee and prior review and documented approval/favorable opinion of the amendment from the relevant Ethics Committee, except where it is necessary to eliminate an immediate hazard to trial patients, or where the change(s) involves only logistical or administrative aspects of the trial (e.g., change in monitor(s), change of telephone number(s)).
Protocol amendments will be submitted to the appropriate authority(ies) as required by the applicable regulatory requirement(s).

13.2 Disclosure of all Information and Results

In signing the final protocol, every participating investigator agrees to keep all information and results concerning the study and the investigational product confidential for as long as the data remain unpublished. The confidentiality obligation applies to all personnel involved at the investigational site.
Publications are in the responsibility of the Study Committee. All publications or presentations of so far undisclosed data must be submitted to Pfizer Deutschland GmbH at least 45 days prior to such submission for publication or presentation.
It is understood that the purposes of such prior submission are:

1. To provide Pfizer Deutschland GmbH with the opportunity to review and comment on the contents of the manuscript or presentation;
2. To permit the Pfizer Deutschland GmbH to file patent applications for any inventions or discoveries contained in such proposed publication or presentation in order to prevent the inadvertent forfeiture of potential world-wide patent rights.

If the proposed publication or presentation is submitted to Pfizer Deutschland GmbH at least 45 days before the proposed submission for publication or presentation date, then the investigator/institution’s right to submit the proposed publication or presentation for publication shall not be compromised by the failure of Pfizer Deutschland GmbH to file patent application(s) in a timely manner.
If a manuscript of the trial results is planned, the Study Committee will co-ordinate the publication with trial participants.

13.3 Premature Discontinuation of the Study in a Trial Site

The Study Committee may stop this trial in one particular site for any of the following reasons:
- The site cannot include an adequate number of patients.
- Serious and/or persistent non-compliance with the protocol.
- Careless or premeditated false documentation in the CRF.
- Inadequate co-operation with the Study Committee, Profil GmbH or its representatives.
- Non-compliance with GCP and/or regulatory requirements.
- The investigator requests discontinuation.

If the trial is prematurely terminated or suspended for any reason, trial patients will be informed promptly, appropriate therapy and follow-up for patients will be assured and, where required by the applicable regulatory requirement(s), the relevant regulatory authority(ies) will be informed. The ethics committees will be informed promptly and provided with a detailed written explanation for the termination or suspension.

13.4 Premature Discontinuation of the Complete Study

The study will be discontinued prematurely in the following cases:
- If undesirable effects that occur are so serious that the benefit-risk ratio is not acceptable.
- If the number of drop-outs is so high that correct completion of the study cannot realistically be expected.
- If a clinically relevant and statistically significant difference for the primary efficacy endpoint can be assessed at the interim analysis, as described in section 9.9).

If the trial is prematurely terminated or suspended, Profil will inform promptly the investigators/institutions, and the regulatory authority(ies) of the termination or suspension and the reason(s) for the termination or suspension. The Ethics Committee will also be informed promptly and provided with the reason(s) for the termination or suspension, as specified by the applicable regulatory requirement(s).

13.5 Insurance for Patients

Profil GmbH will provide the insurance for the subjects participating in this study.

13.6 Contracts, Finances

In addition to the protocol trial-related duties, functions and financial aspects have to be specified in a separate contract between the Study Committee (i.e. the responsible institution) and Profil GmbH, the Study Committee and the Principal Investigator, as well as any other parties involved with the clinical trial.

14 REPORTING

A final integrated clinical/statistical report will be prepared by Profil and reviewed by the Study Committee.

15 REFERENCES

1. Bougneres P, Carel J: Factors associated with early remission of type 1 diabetes in children treated with cyclosporine. *N Eng J Med* **318**: 663-670, 1988.
2. Stiller CR, Dupre J, Gent M, Jenner MR, Keown PA, Laupacis A, Martell R, Rodger NW, von Graffenried B, Wolfe BM: Effects of cyclosporine immunosuppression in insulin-dependent diabetes mellitus of recent onset. *Science* **223**:1362-1367, 1984.
3. Raz I, Elias D, Avron A, Tamir M, Metzger M, Cohen IR: Beta-cell function in new-onset type 1 diabetes and immunomodulation with a heat-shock protein peptide (DiaPep277): a randomised, double-blind, phase II trial. *Lancet* **358**:1749-53, 2001.
4. Herold KC, Hagopian W, Auger JA, Poumian-Ruiz E, Taylor L, Donaldson D, Gitelman SE, Harlan DM, Xu D, Zivin RA, Bluestone JA: Anti-CD3 monoclonal antibody in new-onset type 1 diabetes mellitus. *N Engl J Med* **346**:1692-98, 2002.
5. Kwak B, Mulhaupt F, Myit S, Mach F. Statins as a newly recognized type of immunomodulator. *Nat Med* **6**:1399-402, 2000.
6. Weitz-Schmidt G, Welzenbach K, Brinkmann V, Kamata T, Kallen J, Bruns C, Cottens S, Takada Y, Hommel U: Statins selectively inhibit leukocyte function antigen-1 by binding to a novel regulatory integrin site. *Nat Med.* **7**: 687-92, 2001.
7. Youssef S, Stuve O, Patarroyo JC, Ruiz PJ, Radosevich JL, Hur EM, Bravo M, Mitchell DJ, Sobel RA, Steinman L, Zamvil SS: The HMG-CoA reductase inhibitor, atorvastatin promotes a Th2 bias and reverses paralysis in central nervous system autoimmune disease. *Nature* **420**:78-84, 2002.
8. Aktas O, Waiczies S, Smorodchenko A, Dorr J, Seeger B, Prozorovski T, Sallach S, Endres M, Brocke S, Nitsch R, Zipp F: Treatment of relapsing paralysis in experimental encephalomyelitis by targeting Th1 cells through atorvastatin. *J Exp Med* **197**:725-33, 2003.
9. S. Martin, N. van den Engel, A. Vinke, E. Heidenthal, B. Schulte, H. Kolb: Dominant role of intercellular adhesion molecule-1 in the pathogenesis of autoimmune diabetes in nobese diabetic mice. *J Autoimmunity* **17**:109-117, 2001.
10. van de Ree MA, Huisman MV, Princen HM, Meinders AE, Kluft C: Strong decrease of high sensitivity C-reactive protein with high-dose atorvastatin in patients with type 2 diabetes mellitus. *Atherosclerosis* **166**:129-35, 2003.
11. Greenbaum CJ, Harrison LC: Guidelines for intervention trials in subjects with newly diagnosed type 1 diabetes. *Diabetes* **52**: 1059-1065, 2003.
12. Report of the expert committee on the diagnosis and classification of diabetes mellitus. *Diabetes Care* **26**, 5-20, 2003.
